# Anion–Diluent Decoupled Solvation Chemistry in Ionic Liquid-Based Localized High-Concentration Electrolytes Toward High-Voltage Lithium Metal Batteries

**DOI:** 10.1007/s40820-026-02242-4

**Published:** 2026-06-01

**Authors:** Guangye Wu, Haifeng Tu, Zhicheng Wang, Yiwen Gao, Peng Ding, Yi Yang, Lingwang Liu, Suwan Lu, Farwa Mushtaq, Guochao Sun, Hexiang Chen, Haiyang Zhang, Jiangyan Xue, Jingjing Xu, Hong Li, Xiaodong Wu

**Affiliations:** 1 School of Materials Science and Engineering, Shanghai University, Shanghai, 200444 People’s Republic of China; 2 i-Lab, Suzhou Institute of Nano-Tech and Nano-Bionics, Chinese Academy of Sciences, Suzhou, 215123 Jiangsu People’s Republic of China; 3 School of Nano-Tech and Nano-Bionics, University of Science and Technology of China, Hefei, 230026 Anhui People’s Republic of China; 4 Tianmu Lake Institute of Advanced Energy Storage Technologies Co., Ltd., Liyang, 213300 People’s Republic of China; 5 Beijing Advanced Innovation Center for Materials Genome Engineering Key Laboratory for Renewable Energy, Beijing Key Laboratory for New Energy Materials and Devices, Institute of Physics, Chinese Academy of Sciences, Beijing, 100190 People’s Republic of China; 6 College of Material Science and Engineering, Hohai University, Changzhou, 213022 Jiangsu People’s Republic of China

**Keywords:** Lithium metal batteries, Ionic liquid, Localized high-concentration electrolytes, Solvation structure, Diluent, High-voltage LMBs

## Abstract

Ionic liquid-based localized high-concentration electrolytes (IL-based LHCEs), derived from non-solvating diluents and ionic liquid electrolytes (ILEs), are promising candidates for long-life and safe lithium metal batteries (LMBs). However, conventional understanding holds that diluents merely reduce electrolyte viscosity and enhance ionic conductivity at the macroscopic level, overlooking their influence on the solvation structure at the molecular scale. Herein, we propose an anion–diluent decoupled solvation structure that is more conducive to stable cycling of high-voltage LMBs. Specifically, diluents with weak interactions toward FSI^−^ anions effectively promote coordination between FSI^−^ anions and Li^+^, leading to a solvation structure dominated by contact ion pairs (CIPs). The small anionic clusters within CIPs in IL-based LHCEs further facilitate Li^+^ ion transport. Moreover, compared to aggregate (AGG)-dominated solvation structures rich in anions and electrons, the CIPs exhibit superior oxidation resistance, contributing to the formation of a thin and compact cathode electrolyte interphase (CEI). As a proof, an IL-based LHCE incorporating 1,1,2,2-tetrafluoroethyl methyl ether (TFE) as the diluent (TFE-LHCE) was developed. A Li||TFE-LHCE||NCM523 (LiNi_0.5_Mn_0.3_Co_0.2_O_2_) cell cycled at 4.3 V achieves a capacity retention of 70% after 600 cycles, while a Li||NCM811 (LiNi_0.8_Mn_0.1_Co_0.1_O_2_) cell at 4.5 V retains 88% capacity after 200 cycles. Furthermore, a 2.6 Ah Li||NCM83 (LiNi_0.83_Mn_0.1_Co_0.07_O_2_) pouch cell demonstrates stable cycling over 40 cycles and successfully passes a nail penetration safety test. This work elucidates the critical mechanism by which non‑solvating diluents reconstruct the Li^+^ ion solvation structure, establishing a theoretical foundation for the rational screening and design of high‑performance electrolyte diluents.

**Supplementary Information:**

The online version contains supplementary material available at 10.1007/s40820-026-02242-4.

## Highlights

- Weak diluent-anion interactions decouple FSI⁻ from diluent molecules, promoting a contact ion pair (CIP)-dominant solvation structure in IL-based localized high-concentration electrolytes.
- The CIP-dominant solvation sheath exhibits intrinsically higher oxidation stability and facilitates faster Li⁺ desolvation and interfacial charge transfer kinetics.
- The designed TFE-LHCE enables 600 stable cycles for 4.3 V Li||NCM523 cells and 200 cycles for 4.5 V Li||NCM811 cells, with a 2.6 Ah pouch cell passing nail penetration tests.

**Supplementary Information:**

The online version contains supplementary material available at 10.1007/s40820-026-02242-4.

## Graphical abstract

**Figure:**
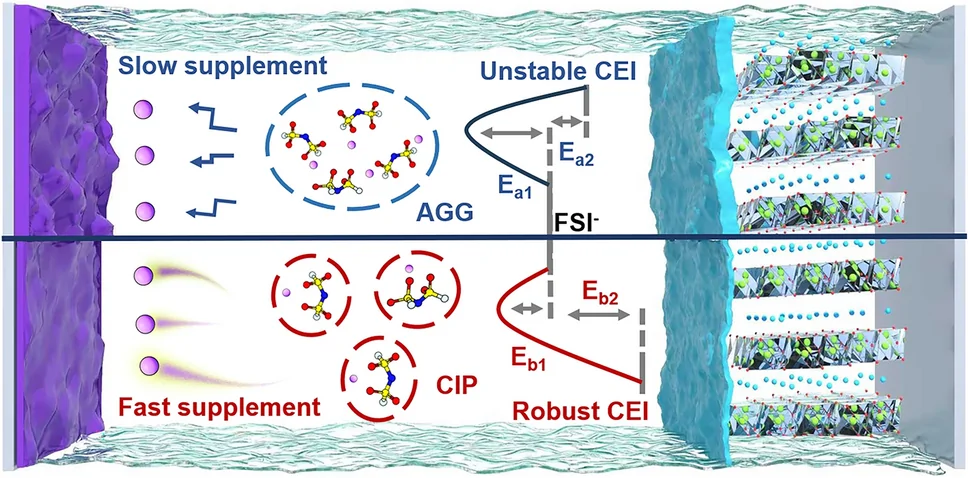

## Introduction

Integrating dendrite-free lithium metal anodes (LMAs) with high-voltage cathodes is a pivotal strategy for constructing high-energy–density lithium metal batteries (LMBs) [1]. However, this battery system still faces formidable challenges in practical applications. On one hand, the extremely low reduction potential of LMAs (− 3.04 V vs. standard hydrogen electrode) renders it susceptible to side reactions with the electrolyte, leading to the formation of an unstable and persistently growing solid electrolyte interphase (SEI) [2–4]. On the other hand, the highly reactive nature of high-nickel cathodes under high-voltage operation triggers oxidative decomposition of the electrolyte [5, 6]. Taking conventional carbonate-based and ether-based electrolytes as examples, the former offers good high-voltage stability but reacts continuously with LMAs, resulting in the constant depletion of active Li metal and electrolyte [7]. While the latter exhibits excellent compatibility with LMAs, its weak oxidation resistance makes it unsuitable for the high-potential window required by high-voltage cathodes [8]. Ionic liquids (ILs), as room-temperature molten salts, are regarded as promising electrolyte candidates for LMBs owing to their high electrochemical stability, extremely low vapor pressure, and outstanding thermal stability [9–11]. Nevertheless, the high cost of ILs, often termed "liquid gold" combined with their high viscosity, which leads to sluggish ion transport kinetics, and hinders their practical application [12]. Thus, achieving the efficient and cost-effective utilization of ILs in electrolyte systems represents a critical challenge.

Hybrid electrolyte systems formed by blending traditional ester or ether solvents with ILs demonstrate certain advantages in reducing viscosity, enhancing ionic conductivity, and lowering costs. For instance, Forsyth et al. reported an ether-assisted IL electrolyte (ILE) by incorporating 20 wt% 1, 2-dimethoxyethane (DME) solvent into a high-concentration ILE to improve overall performance [13]. In our previous work, we also proposed using dimethyl carbonate (DMC) as a co-solvent to formulate a high-concentration dual-anion ILE for high-voltage LMBs [14]. However, such hybrid systems reliant on conventional organic solvents remain constrained by the inherent limitations of the solvents themselves, making it difficult to simultaneously enhance kinetic performance while preserving electrochemical stability, interfacial quality, and battery safety. The design strategy of IL-based localized high-concentration electrolytes (LHCEs) offers a novel approach to resolving these contradictions. This system is constructed by introducing a diluent that does not participate in first Li^+^ ion solvation structure [15, 16]. The diluent in LHCE preserves the local solvation sheath, thereby maintaining the high interfacial stability of ILE while significantly improving its wettability and Li^+^ transport kinetics. Numerous studies have confirmed that fluorinated compounds such as 1,1,2,2-tetrafluoroethyl 2,2,3,3-tetrafluoropropyl ether (TTE) [17], bis(2,2,2-trifluoroethyl) ether (BTFE) [18], and trifluoromethoxybenzene (BnOCF) [19] can serve as effective diluents for IL-based LHCEs.

However, a significant knowledge gap persists regarding the rational design of diluents for IL-based LHCEs. Conventional perspectives often simplistically regard fluorinated ether diluents as inert components, assuming they merely physically separate Li^+^ ion solvation structures [20–22]. This model fails to explain why identical salt and ILs systems exhibit vastly different solvation behaviors and battery performance under the influence of different diluents, particularly regarding notable disparities in Li deposition morphology and high-voltage cathode stability. Recent research suggests that diluents may exhibit complex pseudo-inert behavior. For example, monofluorobenzene (mFBn), with single fluorine functional group, can partially solvate Li^+^, weakening Li^+^-FSI^−^ interactions and consequently increasing ionic conductivity [23]. However, even when diluents themselves show no apparent tendency to coordinate with Li^+^ ions, IL-based LHCEs modulated by different diluents still display distinct performance variations. Without a profound understanding of the role of diluent molecules at the solvation structure level, the targeted development of high-performance IL-based LHCEs will encounter substantial bottlenecks.

In this work, using two structurally similar but non-solvating diluents, 1,1,2,2-tetrafluoroethyl methyl ether (TFE) and 1,2-bis(1,1,2,2-tetrafluoroethoxy)ethane (TFEE) as model systems, we propose a solvation structure based on anion–diluent decoupled solvation structure in IL-based LHCE. Although fluorinated diluents do not directly enter the Li^+^ ion solvation sheath, the diluent molecules can form ion–dipole interactions and hydrogen-bonding interactions with FSI^−^ anions. Specifically, the strong interaction between TFEE diluent molecules and FSI^−^ anions leads to a transfer of negative charge from the FSI^−^ anions to the TFEE molecules, thereby weakening their coordination ability with Li^+^. To effectively neutralize Li^+^, a larger number of FSI^−^ anions are required to participate in the coordination, which facilitates the formation of an aggregate networks (AGGs). This AGG structure is unfavorable for the efficient transport of Li^+^ ions, and FSI^−^-enriched AGG structure exhibits poorer oxidation stability compared to the anion-deficient contact ion pairs (CIPs), resulting in excessive oxidation of FSI^−^ anions and the formation of a thicker, higher-impedance cathode electrolyte interphase (CEI). In contrast, TFE interacts more weakly with FSI^−^, favoring the direct formation of CIPs structure. This CIP-dominated solvation structure features smaller ion cluster sizes, superior transport kinetics, and enhanced oxidation resistance. As a proof, the IL-based LHCE employing TFE as the diluent (TFE-LHCE) demonstrates exceptional reversibility in Li deposition/stripping. The Li||TFE-LHCE||Cu cell maintains an average Coulombic efficiency as high as 99% over 600 stable cycles, and the Li||TFE-LHCE||Li cell can also operate continuously for 1200 h. Furthermore, TFE-LHCE delivers outstanding performance in high-voltage LMBs. The 4.3 V Li||NCM523 (LiNi_0.5_Mn_0.3_Co_0.2_O_2_) battery achieves a capacity retention (CR) of 70% after 600 cycles, and the 4.5 V Li||NCM811 (LiNi_0.8_Mn_0.1_Co_0.1_O_2_) battery retains 88% CR after 200 cycles. A 2.6 Ah Li||NCM83 (LiNi_0.83_Mn_0.1_Co_0.07_O_2_) pouch cell stably cycles for 40 cycles and successfully passes the nail penetration safety test. This study elucidates the fine-tuning mechanism of non-solvating diluents on the solvation structure of IL-based LHCEs, providing a new theoretical foundation for the design of next-generation high-voltage electrolytes.

## Experimental Details

### Materials Preparation and Batteries Assemble

Molecular sieves (3 Å) were activated in a muffle furnace at 300 °C for 72 h. N‑methyl‑N‑propylpyrrolidinium bis(fluorosulfonyl)imide (Pyr_13_FSI) (99.9%, Changde Dadu New Materials Co., Ltd.) and lithium bis(fluorosulfonyl)imide (LiFSI) (99.9%, Duoduo Chemical Co., Ltd.) were dried at 100 °C for 24 h before use. 1,1,2,2‑Tetrafluoroethyl methyl ether (TFE) (Adamas, 99.8%) and 1,2‑bis(1,1,2,2‑tetrafluoroethoxy)ethane (TFEE) (Adamas, 99.8%) were activated with molecular sieves at room temperature for 48 h before use. Lithium foil with a thickness of 450 μm was purchased from China Energy Lithium Co., Ltd. Cathodes with NCM523 loading (11.46 mg cm^−2^) and NCM811 loading (9.97 mg cm^−2^) were obtained from Shenzhen Kejing Technology Co., Ltd. Battery assembly was conducted in an argon-filled glove box. All electrochemical tests were performed at 25 °C. CR2025-type coin cells were assembled to evaluate the electrochemical performance of the electrolyte. All coin cells used a lithium foil with a thickness of 450 µm and a diameter of 15.6 mm as the anode, and 75 µL of electrolyte was added to each cell.

### Characterizations

Fourier transform infrared (FTIR) spectroscopy (Thermo Scientific Nicolet 6700 spectrometer) were used to investigate the solvation structures. Raman spectroscopy (LABRAM, HR) was tested with 532 nm laser excitation. The microstructure of plated Li surface was studied by a cold cathode field emission scanning electron microscopy (SEM, Hitachi, S4800, Japan). In addition, the information of the solid electrolyte interphase (SEI) and cathode electrolyte interphase (CEI) was studied by X-ray photoelectron spectroscopy (XPS, Thermo Scientific K-Alpha +), which was conducted on a spectrometer (72 W and 12 kV) with an Al Kα source. The component distribution of the SEI layer was further characterized by time-of-flight secondary ion mass spectrometry (IONTOF TOF.SIMS 5–100), which was equipped with a 1 keV Cs + sputter gun and an electron flood gun for charge neutralization. The Li anode samples were washed by DMC and dried in an argon glovebox, then transferred into the SEM, XPS, and TOF–SIMS analysis chamber by a vacuum transfer device to avoid contact with air and water. The TEM images of CEI layers were acquired on a Hitach HT-7700 transmission electron microscope with an accelerating voltage of 120 kV. Nail penetration test of the Li||Ni83 pouch cells was performed by a battery extrusion needle testing machine (BE-6047-50T), a nail with diameter of 5–8 mm was driven into the pouch cells at a rate of 25 ± 5 mm s^−1^. Wide-angle X-ray scattering (WAXS) was conducted on Xeuss 3.0 to probe solvation structure.

### Electrochemical Measurements

The ionic conductivity of the electrolyte was measured using a Mettler Toledo S400 SevenExcellence instrument equipped with an InLab 741-ISM conductivity electrode. The electrochemical impedance spectroscopy (EIS) of Li||Li coin cells was measured using a VMP-300-based instrument with a booster (BioLogic). The distribution of relaxation times (DRT) plots was derived from the EIS data and subsequently analyzed using MATLAB software. Coulombic efficiency (CE) was evaluated through lithium deposition/stripping cycles on Cu foil. Prior to cycling, a formation cycle was performed: 5 mAh cm^−2^ of Li was deposited on the Cu substrate, followed by stripping at a current density of 0.5 mA cm^−2^ until reaching a cutoff voltage of 1 V. Subsequently, 5 mAh cm^−2^ of Li was deposited on the Cu electrode at 0.5 mA cm^−2^ to establish a Li reservoir, followed by 10 repeated stripping/deposition cycles of 1 mAh cm^−2^ Li (stripping current density: 0.5 mA cm^−2^, deposition current density: 0.5 mA cm^−2^). Finally, the remaining Li metal after the 10 cycles was stripped at 0.5 mA cm^−2^ until reaching 1 V. The average CE was obtained by dividing the total stripping capacity by the total deposition capacity after the initial formation cycle.

### Computational Methods

Molecular dynamics simulations were performed using the Forcite module of Materials Studio 2020. First, based on the specific molecular ratio of the electrolyte, the amorphous cell module was employed to construct the electrolyte model by placing LiFSI, Pyr_13_FSI, and diluent molecules into a cubic simulation box. The COMPASS III force field was used to calculate the intermolecular interactions. Initially, the simulation was conducted under the NPT ensemble at 298.15 K and 1.0 × 10^–4^ GPa to minimize the system energy. During this stage, temperature was controlled using the Nosé-Hoover method, with a van der Waals cutoff radius set to 12.5 Å. Subsequently, an NVT simulation was performed at 298.15 K. The simulation durations for the NPT and NVT phases were 0.5 and 1 ns, respectively, both with a time step of 1 fs. The entire NVT simulation trajectory was used for analyzing the solvation structure. The Multiwfn 3.8 program was employed to derive restrained electrostatic potential (RESP) atomic charges based on high‑quality electronic wave functions obtained from quantum chemical calculations [24, 25]. These calculations were performed using Gaussian16 software at the level of the B3LYP‑D3 functional and the def2‑SVP basis set [26, 27].

## Results and Discussion

### Diluent–Anion Interactions Dictate Solvation Structure in IL-Based LHCEs

**Fig. 1:**
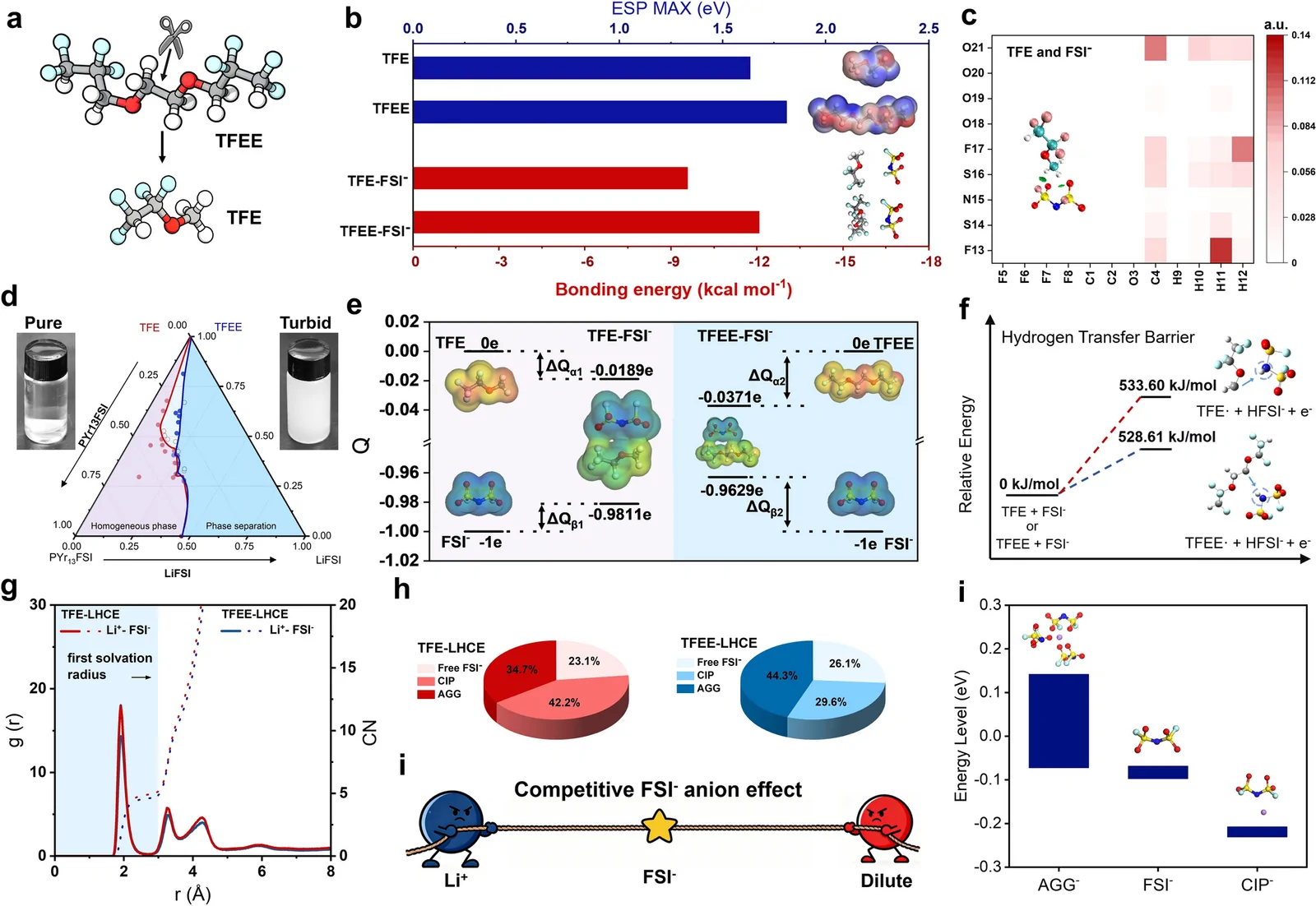
**a** Molecular structure of TFE and TFEE diluents. **b** Maximum electrostatic potential values of the two diluents, and their binding energies with the FSI^−^ anion. **c** Intermolecular contact matrix and interaction energy gradient isosurfaces for the TFE-FSI^−^ pair obtained through the reduced density gradient (RDG) method. The a.u. on the coordinate axis represents the electron overlap density between two atoms. The larger the value, the stronger the interaction between the two atoms and the closer their distance. **d** Ternary phase diagrams of the LiFSI/Pyr_13_FSI ionic liquid system with the TFE and TFEE diluents, respectively. **e** Calculated charge transfer amounts between TFE and TFEE molecules and FSI^−^ anions. **f** Activation energy for α-H transfer in two different diluents. **g** RDF and corresponding CN for Li^+^-FSI^−^ in the TFE-LHCE and TFEE-LHCE systems, calculated based on molecular dynamics simulations. **h** Proportions of various solvation structures in the TFE-LHCE and TFEE-LHCE electrolyte systems. **i** Schematic illustration of the competition between Li^+^ cations and diluent molecules for coordination with the FSI^−^ anion. **j** Calculated HOMO and LUMO values for different electrolyte structures

To investigate the influence of different diluent molecules on IL-based LHCEs, two hydrofluoroethers with similar molecular structures 1,1,2,2-tetrafluoroethyl methyl ether (TFE) and 1,2-bis(1,1,2,2-tetrafluoroethoxy)ethane (TFEE) were selected, as shown in Fig. 1a, where TFE can be regarded as half of the TFEE molecule. As shown in Fig. 1b, the electrostatic potentials (ESP) of the diluent molecules were calculated using density functional theory (DFT). The results revealed that the ESP_max_ of TFEE (1.811 eV) is higher than that of TFE (1.635 eV), indicating that TFEE is more prone to ion–dipole interactions with the negatively charged FSI^−^ anion [28–30]. Additionally, the binding energy between the FSI^−^ anion and TFEE (-12.076 kcal mol^−1^) is higher than that between FSI^−^ and TFE (-9.576 kcal mol^−1^). Further independent gradient model based on Hirshfeld partition (IGMH) analysis demonstrated that hydrogen-bonding interactions can also form between the hydrofluoroether diluents and FSI^−^ by δH(diluent)–δF(FSI^−^), as shown in Figs. 1c and S1-S2 [31, 32]. This interaction was further confirmed by the ^1^H NMR spectra of the pure diluents and their mixtures with Pyr_13_FSI (Fig. S3). Upon mixing, the proton peak of the difluoromethyl group (-CF₂H) in TFEE exhibits a larger downfield shift (ΔX₁) compared to that of TFE (ΔX₂). This greater deshielding in TFEE is direct evidence of a stronger hydrogen-bonding interaction (δH–δF) and ion–dipole interaction with the electron-rich FSI⁻ anion.

The strength of interactions among different species in the electrolyte solution directly reflects their mutual solubility, with stronger interactions generally leading to higher miscibility. To experimentally validate the interactions between diluent molecules and FSI^−^ anions, ternary phase diagrams of the diluent, Pyr_13_FSI, and LiFSI were constructed, as shown in Fig. 1d. The left side of the phase diagram represents the miscible region, while the right side indicates the immiscible region. The TFEE-Pyr_13_FSI-LiFSI system exhibits a larger miscible region compared to the TFE-Pyr_13_FSI-LiFSI system. Additionally, when electrolytes were prepared with a molar ratio of LiFSI:Pyr_13_FSI:diluent = 1: 2: 3, the system containing TFEE as the diluent appeared clear (left inset in Fig. 1d), whereas the system containing TFE appeared turbid (right inset in Fig. 1d). Therefore, the TFE diluent molecule, due to its lower ESP_max_ and weaker interaction with FSI^−^ anions, is more prone to phase separation in LHCEs, which is consistent with the findings reported by Fan et al. [33]. The charge transfer from FSI^−^ to TFE and TFEE was quantitatively calculated, as shown in Fig. 1e. The FSI^−^ anion transfers 0.0189 e to TFE and 0.0371 e to TFEE. The energy barriers for α-H transfer from TFE and TFEE to FSI^−^ are 533.60 and 528.61 kJ mol^−1^, respectively, suggesting that the TFE-FSI^−^ complex exhibits higher oxidation stability compared to the TFEE-FSI^−^ complex [34].

The strength of interactions between hydrofluoroether diluent molecules and FSI^−^ anions indirectly affects the solvation structure of the electrolyte, despite their non-solvating characteristics. Two different electrolyte formulations including LiFSI/Pyr_13_FSI/TFE (1:2:2 by mol) LHCE (denoted as TFE-LHCE) and LiFSI/Pyr_13_FSI/TFEE (1:2:2 by mol) LHCE (denoted as TFEE-LHCE) were prepared. Molecular dynamics simulations (MD) were employed to analyze the solvation structures of the TFE-LHCE and TFEE-LHCE systems (Fig. S4) [35]. Radial distribution function (RDF) and coordination number (CN) analyses revealed no interactions between Li^+^ and the F atoms in TFE or TFEE, confirming the non-solvating diluent characteristics of TFE and TFEE (Fig. S5). Notably, the coordination number of Li^+^ with O atoms in FSI^−^ is 5.02 in the TFE-LHCE system, which is higher than the 4.68 in the TFEE-LHCE system. Similarly, the coordination number of Li^+^ with the entire FSI^−^ anion is 5.03 in TFE-LHCE, also higher than the 4.71 in TFEE-LHCE (Fig. 1g). In the TFE-LHCE system, the proportions of free FSI^−^, CIPs, and AGGs are 23.1%, 42.2%, and 34.7%, respectively, with the solvation structure dominated by CIPs. In contrast, the TFEE-LHCE system exhibits proportions of 25.8%, 29.1%, and 45.1% for free FSI^−^, CIPs, and AGGs, respectively, with the solvation structure dominated by AGGs (Fig. 1h). The interaction between Li^+^ and FSI^−^ is governed by Coulombic forces, while the interaction between FSI^−^ and diluent molecules includes ion–dipole interactions and typical δH(diluent)–δF(FSI^−^) hydrogen-bonding interactions. A competitive coordination mechanism exists between Li^+^ and diluent molecules for FSI^−^ anions (Fig. 1i). The fundamental reason for the formation of two distinct types of solvation structures lies in the difference in the strength of interactions between FSI^−^ and the diluent molecules. The stronger interaction between the TFEE diluent molecule and the FSI^−^ anion leads to the transfer of negative charge from FSI^−^ to TFEE, thereby weakening its coordination ability with Li^+^. To effectively neutralize the positive charge of Li^+^, a larger number of FSI^−^ anions are required to participate in the coordination, facilitating the formation of an AGG network structure intertwined with multiple FSI^−^ anions. In contrast, the TFE diluent molecule is relatively inert, allowing FSI^−^ to retain its full negative charge and efficiently coordinate with individual Li^+^ ions to form CIPs. Compared to the AGG clusters in TFEE-LHCE, the CIPs structures in TFE-LHCE exhibit smaller solvation cluster sizes, which enhances ionic conductivity and Li^+^ transport kinetics [36]. As shown in Fig. 1i, the electron-rich AGG structure, due to its electron-donating characteristics, exhibits a significantly higher highest occupied molecular orbital (HOMO) value (-0.07229) than CIPs (-0.23075), indicating that the AGG-dominated solvation structure at the cathode side has inferior oxidation stability compared to the anion-deficient CIPs structure.

### Physicochemical Properties and Solvation Structure Characterization

**Fig. 2:**
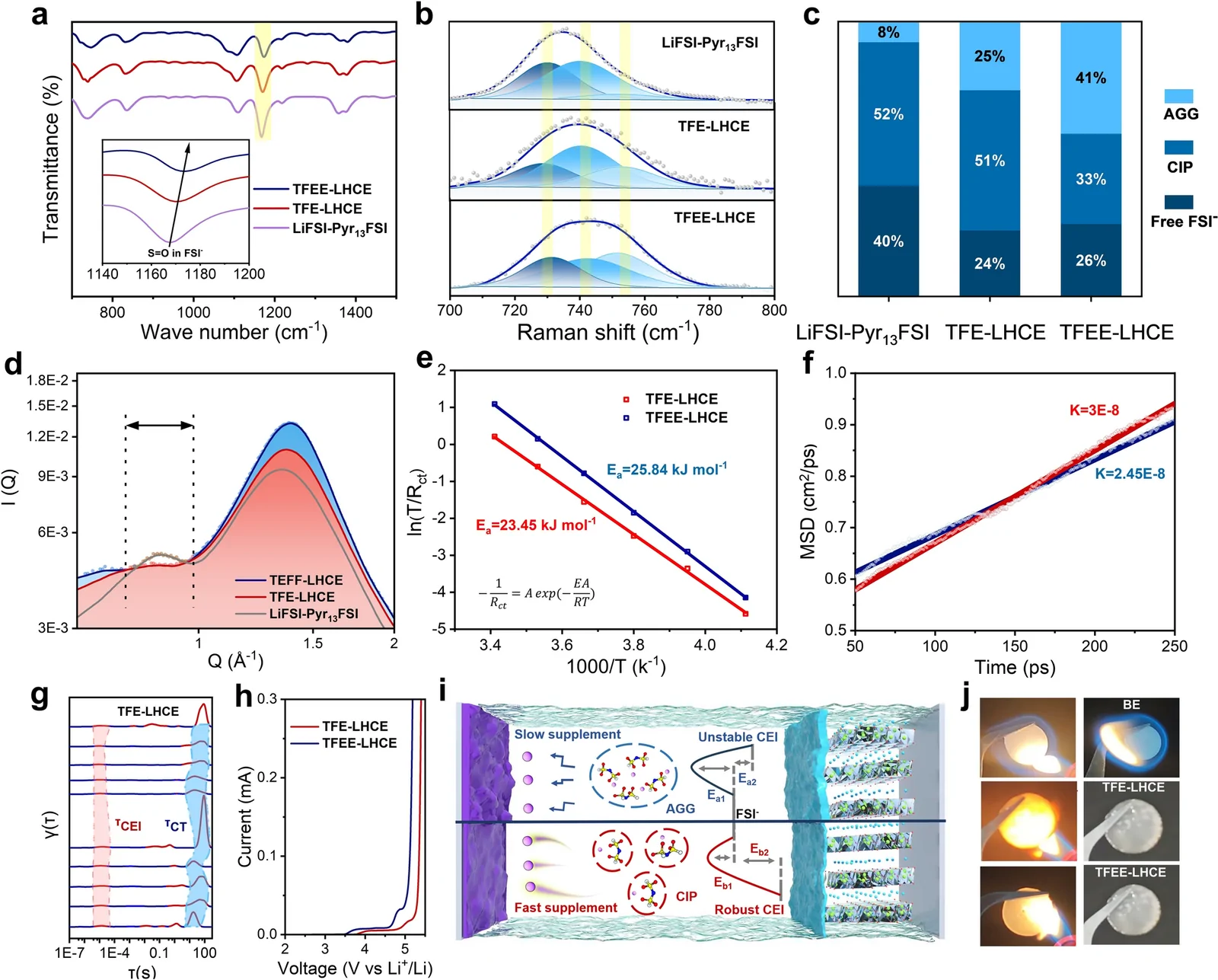
**a** FTIR of TFEE-LHCE, TFE-LHCE, and LiFSI-Pyr_13_FSI. **b** Raman spectra of TFEE-LHCE, TFE-LHCE, and LiFSI-Pyr_13_FSI and **c** corresponding ratios of free FSI^−^, CIPs, and AGGs derived from Raman spectra. **d** WAXS analysis of TFEE-LHCE, TFE-LHCE, and LiFSI-Pyr_13_FSI. **e** Desolvation energy calculated from Arrhenius equation. **f** MSD plots of Li^+^ ions in TFE-LHCE and TFEE-LHCE obtained by MD simulations. **g** DRT analysis derived from in situ EIS of Li||TFE-LHCE||NCM811 cells after formation cycles at a 4.3 V cutoff voltage. **h** Linear sweep voltammetry (LSV) profiles of Li/carbon coated Al cells using TFE-LHCE and TFEE-LHCE with a scan rate of 0.1 mV s^−1^. **i** Schematic illustration of the interfacial chemical changes induced by CIPs and AGGs in Li^+^ ion transport kinetics and oxidation stability. **j** Combustion test of BE, TFE-LHCE, and TFEE-LHCE

Both the thermodynamic stability and the Li^+^ transport kinetics of electrolytes are closely related to their solvation structures [37, 38]. First, Fourier transform infrared spectroscopy (FTIR) was employed to analyze the solvation structures of the different electrolytes [39]. After the addition of TFE and TFEE, the characteristic peaks of the FSI^−^ anion (1170 cm^−1^) exhibited a blue shift, with TFEE showing a more pronounced shift due to its stronger interaction with FSI^−^ (Fig. 2a). Raman spectroscopy was utilized to quantitatively analyze the proportion of various components in the solvation structures of the different electrolytes (Fig. 2b, c). The LiFSI-Pyr_13_FSI system refers to an electrolyte with a LiFSI to Pyr_13_FSI molar ratio of 1:2. In the Raman spectra, peaks at 732, 742, and 752 cm^−1^ correspond to free FSI^−^, CIPs, and AGGs, respectively [40]. Compared to LiFSI-Pyr13FSI, the addition of TFE and TFEE led to a significant reduction in free FSI^−^ content and a corresponding increase in AGGs. Notably, the CIPs content in the TFE-LHCE system (51%) was higher than that in TFEE-LHCE (33%), while its AGG content (25%) was lower than that of TFEE-LHCE (41%). This indicates that TFE-LHCE and TFEE-LHCE form solvation structures dominated by CIPs and AGGs, respectively. To further investigate the microstructural differences between TFE-LHCE and TFEE-LHCE, wide-angle X-ray scattering (WAXS) was conducted (Figs. 2d and S6). The LiFSI-Pyr_13_FSI sample exhibited a characteristic peak in the 0.7–1.0 Å^−1^ range, corresponding to the spacing between FSI^−^ anions. After the introduction of diluents, this peak shifted to lower Q values, indicating that the diluents promote the formation of larger FSI^−^ clusters [41]. Notably, the peak shift was more pronounced for TFEE, suggesting its stronger ability to facilitate anion cluster aggregation. CIP-dominated (TFE-LHCE) and AGG-dominated (TFEE-LHCE) solvation structures exhibit distinct differences in Li^+^ transport kinetics and electrolyte thermodynamic stability. To investigate the influence of different cluster structures on Li^+^ conduction, the impedance of Li||Li symmetric cells was measured at various temperatures, and the Li^+^ desolvation energy was calculated using the Arrhenius equation (Fig. 2e). The Li^+^ desolvation energy for TFE-LHCE was 23.45 kJ mol^−1^, lower than the 25.84 kJ mol^−1^ for TFEE-LHCE. Furthermore, the mean squared displacement (MSD) over 200 ps was simulated to evaluate Li^+^ transport kinetics (Fig. 2f) [42]. The steeper slope of the MSD curve for TFE-LHCE indicates faster Li^+^ migration, consistent with conductivity and Li^+^ transference number measurements. The ionic conductivity of TFE-LHCE was 2.284 mS cm^−1^, higher than the 1.602 mS cm^−1^ for TFEE-LHCE (Fig. S7). The TFE-LHCE electrolyte exhibited a Li^+^ transference number of 0.26, whereas the TFEE-LHCE electrolyte showed a relatively lower value of 0.23 (Fig. S8). Viscosity measurements (Fig. S9) show that the addition of either TFE or TFEE drastically reduces the viscosity of the neat ionic liquid (LiFSI-Pyr_13_FSI). Critically, TFE-LHCE exhibits a higher viscosity than TFEE-LHCE, indicating that the improvement in lithium ion transport properties is attributed to the changes in the microscopic solvation structure, and is less affected by the viscosity of the electrolyte.

Additionally, Li||NCM811 cells were assembled and analyzed using in situ distribution of relaxation times (DRT) to further validate the differences in Li^+^ transport kinetics between the two systems [43]. Peaks in the mid-frequency (10^3^–10 Hz) and low-frequency (10^–7^-10^–4^ Hz) regions correspond to interfacial impedance (R_CT_) and cathode electrolyte interphase (CEI) resistance (R_CEI_), respectively. During cycling, TFE-LHCE exhibited significantly lower τ_CT_ values, again confirming its superior Li^+^ transport kinetics (Figs. 2g and S10). We also performed in situ EIS on Li||Li symmetric cells and transformed the data into DRT plots (Fig. S11). The DRT analysis reveals a significantly lower interfacial charge transfer resistance (R_CT_) for TFE-LHCE compared to TFEE-LHCE during a full deposition/stripping cycle. This directly validates that CIP-dominated solvation structures facilitate faster interfacial kinetics. On the other hand, the electron-deficient CIP-dominated solvation structure demonstrated higher oxidation stability compared to the electron-rich AGG-dominated structure (Fig. 1h). In summary, the smaller average size of anion-deficient CIPs clusters is conducive to superior Li^+^ transport kinetics and is expected to promote dense deposition morphology on the Li metal anode surface. Simultaneously, the CIPs structure exhibits good oxidation stability, which can suppress excessive oxidation of FSI^−^ at the cathode side and facilitate the formation of a thin and dense CEI (Fig. 2i). Furthermore, IL-based LHCEs possess non-flammable characteristics, significantly enhancing battery safety in practical applications compared to conventional carbonate-based electrolytes (Fig. 2j).

### Enhanced Li Metal Anode Compatibility and Interfacial Kinetics

**Fig. 3:**
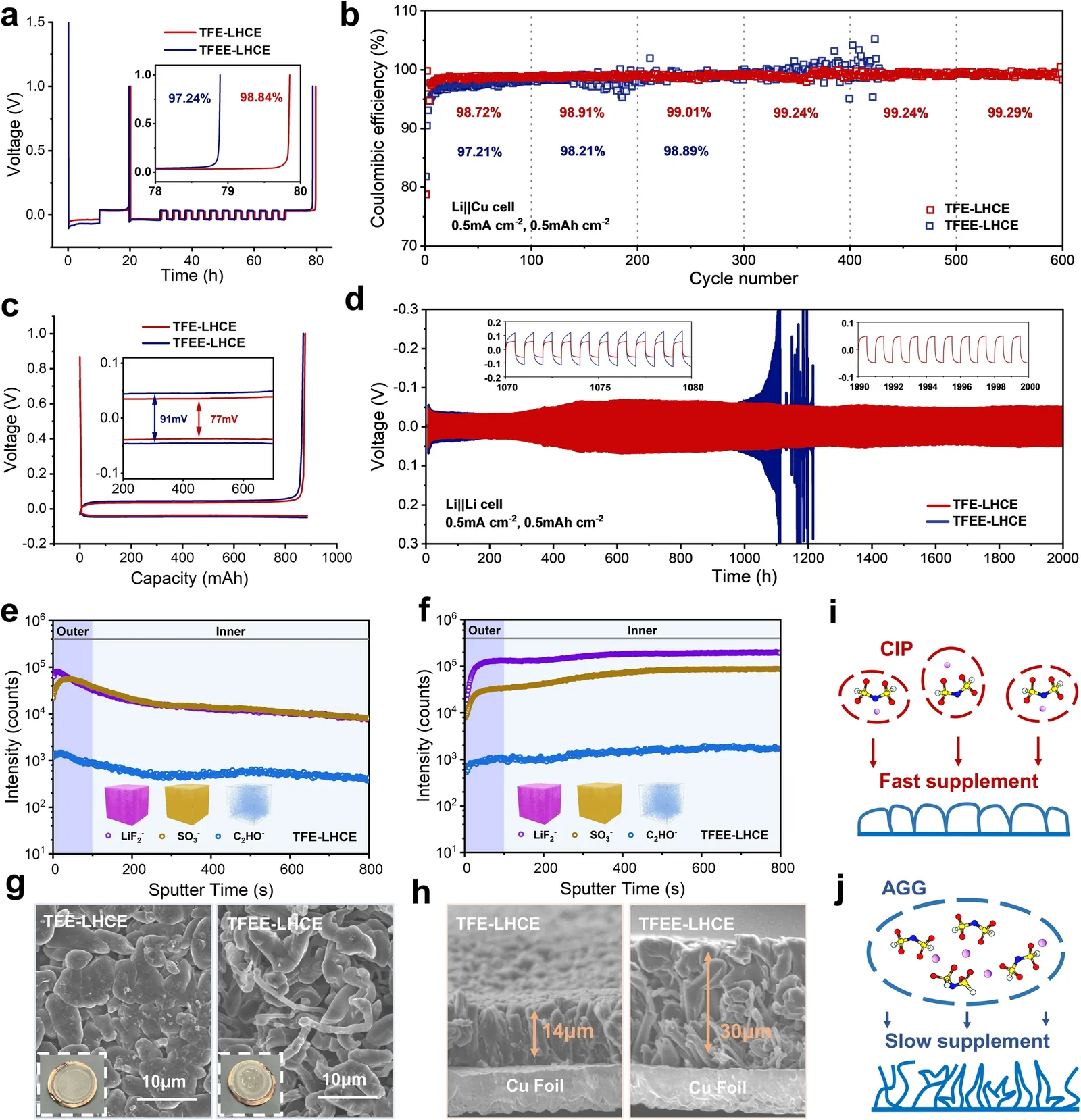
**a** Aurbach CE test of TFEE-LHCE and TFE-LHCE. **b** Coulombic efficiency of Li deposition/stripping in Li||Cu cells using TFE-LHCE and TFEE-LHCE electrolytes at a current density of 0.5 mA cm^−2^ and an areal capacity of 0.5 mAh cm^−2^. **c** Voltage profiles corresponding to Li deposition/stripping in Li||Cu cells. **d** Voltage profiles of Li||Li symmetric cells with TFE-LHCE and TFEE-LHCE at 0.5 mA cm^−2^, 0.5 mAh cm^−2^. TOF–SIMS analysis of SO_2_^−^, C_2_HO^−^, and LiF_2_^−^ species on the cycled Li metal anode extracted from **e** Li||TFE-LHCE||Li and **f** Li||TFEE-LHCE||Li cells. SEM images in **g** top-view and **h** cross-sectional mode for 2.0 mAh cm^−2^ Li deposited on Cu in TFE-LHCE and TFEE-LHCE. Schematic illustration of Li^+^ ion deposition morphologies governed by **i** CIP-dominated solvation structure and **j** AGG-dominated solvation structure

The reversibility of the Li deposition/stripping process directly affects the cycle life and capacity retention of batteries. Therefore, the Coulombic efficiency (CE) of TFE-LHCE and TFEE-LHCE in Li||Cu cells was first evaluated using the Aurbach method (Fig. 3a) [38]. The results show that the CE of the Li||TFEE-LHCE||Cu cell during lithium stripping/deposition is 97.24%, which is lower than that of the Li||TFE-LHCE||Cu cell (98.84%). Further long-term cycling CE tests were conducted at a current density of 0.5 mA cm^−2^ and a deposition capacity of 0.5 mAh cm^−2^. The Li||Cu cell using TFE-LHCE could stably cycle for over 600 cycles, while the cell using TFEE-LHCE exhibited CE fluctuations after approximately 200 cycles and eventually failed around 400 cycles (Figs. 3b and S12). Meanwhile, the average CE per 100 cycles of the Li||TFE-LHCE||Cu cell was consistently higher than that of the TFEE-LHCE system, and its charge/discharge profiles exhibited lower overpotential.

To further investigate, Li||Li symmetric cells were assembled and tested. Under conditions of 0.1 mA cm^−2^ and 0.1 mAh cm^−2^, the TFE-LHCE system showed lower polarization voltage (Fig. S13). When the current density and deposition capacity were increased to 0.5 mA cm^−2^ and 0.5 mAh cm^−2^, the Li||TFE-LHCE||Li cell still exhibited low overpotential and could stably cycle for 2000 h, while the Li||TFEE-LHCE||Li cell showed a significant increase in polarization voltage after approximately 1000 h (Fig. 3d). Compositional analysis was performed on the cycled LMAs. The time-of-flight secondary ion mass spectrometry (TOF–SIMS) results indicate that the interphase components formed by TFE-LHCE and TFEE-LHCE are similar, including species such as LiF_2_^−^, SO_3_^−^, and C_2_HO^−^ (Fig. 3e, f). Notably, as the sputtering time increased, the signals of the components in the SEI formed by TFE-LHCE gradually weakened (Fig. 3e), whereas those formed by TFEE-LHCE gradually intensified (Fig. 3f). This suggests that the SEI layer formed by TFE-LHCE is thinner, while TFEE-LHCE reacts more extensively with the LMA, leading to more severe corrosion. The O 1*s* X-ray photoelectron spectroscopy (XPS) spectra further confirm this observation (Fig. S14), as the Li_2_O component (530 eV) in the TFEE-LHCE system exhibits a more pronounced vertical distribution, consistent with the TOF–SIMS analysis.

The surface and cross-sectional morphologies of Li metal deposited on Cu foil with a capacity of 2 mAh cm^−2^ were observed using scanning electron microscopy (SEM). As shown in Fig. 3g, the deposited Li exhibits a dense and flat block-like morphology, with a smooth and bright surface in optical images when TFE-LHCE was used. In contrast, when TFEE-LHCE was used, the Li surface shows extensive dendrite formation, and black dead Li regions are visible in optical images. In terms of cross-sectional thickness, the deposited Li in TFE-LHCE system is approximately 14 μm, which is close to the theoretical Li thickness of 10 μm for 2 mAh cm^−2^. However, the deposited Li thickness in TFEE-LHCE reaches 30 μm due to significant dendrite growth, indicating severe volume expansion. Further comparison of the morphology after 50 cycles of Li||Li symmetric cells reveals that the Li metal surface in TFE-LHCE remains smooth and dense, whereas TFEE-LHCE results in a loose and porous structure (Fig. S15). In summary, the excellent Li anode compatibility and ideal deposition morphology of TFE-LHCE electrolyte are primarily attributed to its high content of CIPs. As small-sized solvation clusters, CIPs facilitate efficient Li^+^ migration in the bulk electrolyte and continuously supply Li^+^ ions to the interface, thereby promoting the formation of a smooth and dense Li deposition layer (Fig. 3i). In contrast, within the AGG-dominated solvation structure, the larger anion clusters substantially increase the kinetic barrier for Li^+^ transport, resulting in sluggish Li^+^ stripping/deposition processes at the Li metal surface and the subsequent formation of loose and porous dendritic Li morphologies. This unstable Li^+^ deposition morphology fails to effectively prevent continuous side reactions between the electrolyte and the LMAs (Fig. 3j).

### Oxidative Stability and CEI Evolution

**Fig. 4:**
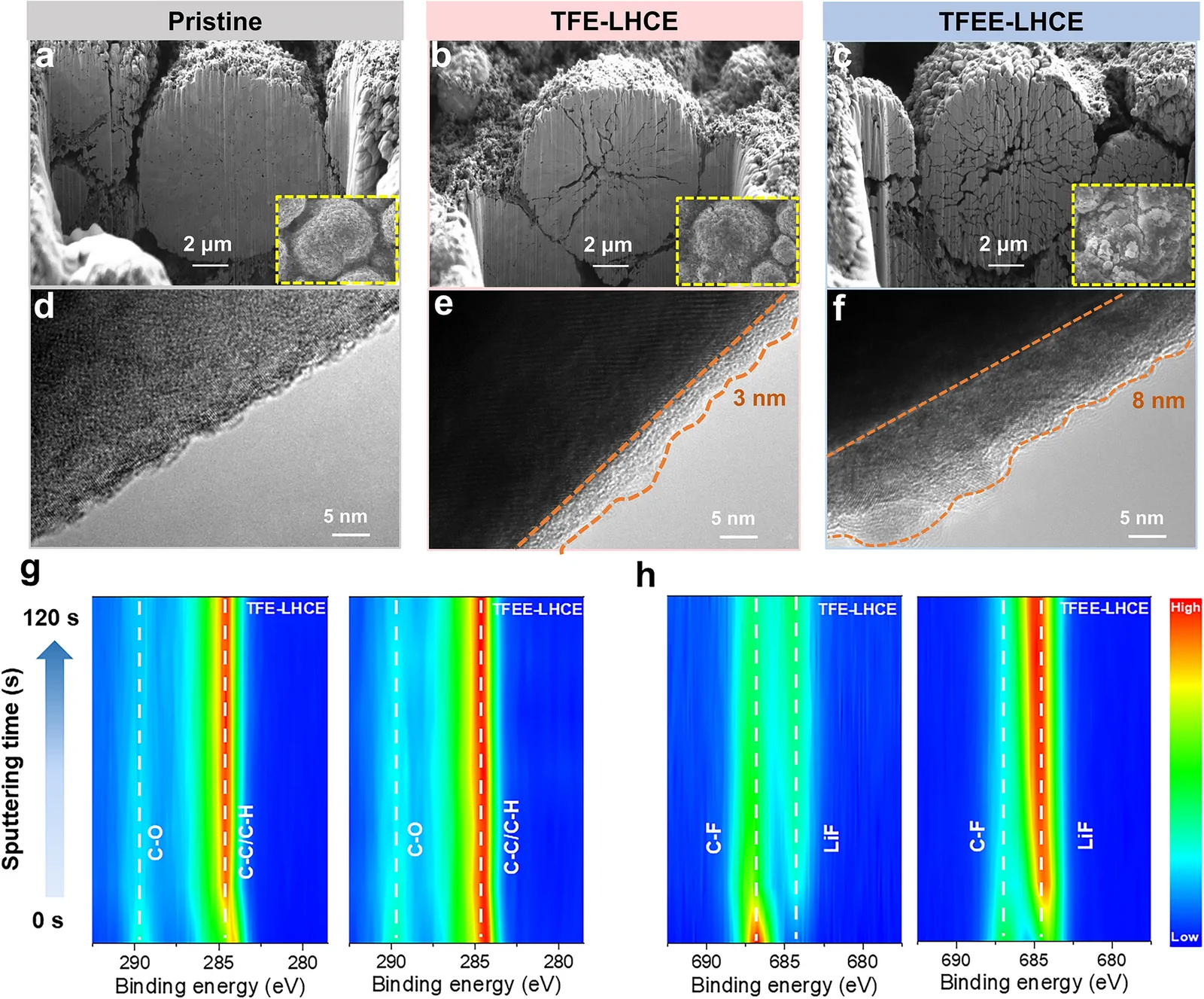
**a** FIB–SEM cross-sectional images of the pristine **a** cathode and the post-200-cycle NCM811 secondary spherical particles in **b** TFE-LHCE and **c** TFEE-LHCE electrolytes, with the inset showing the surface morphology of the spherical particles. TEM characterization of the morphology and thickness of the SEI layers formed on **d** the pristine cathode surface and the NCM811 surfaces in **e** LiFSI-TFE and **f** LiFSI-TFEE electrolytes. Contour plots of **g** C 1*s* and **h** F 1*s* for the cycled Li anode obtained from XPS spectra at different sputtering times

On the cathode side, we investigated the effects of TFE-LHCE and TFEE-LHCE electrolytes on the structural evolution and CEI morphology of polycrystalline LiNi_0.8_Co_0.1_Mn_0.1_O_2_ (NCM811) cathodes after 200 cycles in Li||NCM811 cells. The pristine polycrystalline NCM811 cathode particles exhibit an intact structure with a smooth surface (Fig. 4a, d). After cycling with TFE-LHCE, microcracks appear in the secondary particles, and uniformly covered decomposition products are observed on the surface of primary particles (Figs. 4b and S16). Transmission electron microscopy (TEM) characterization reveals that TFE-LHCE forms a CEI layer approximately 3 nm thick on the cathode surface (Fig. 4e). In contrast, significant intergranular cracks are observed in NCM811 cathode particles after cycling with TFEE-LHCE. This is primarily attributed to the excessive decomposition of the anion-rich AGG solvation structure in TFEE-LHCE at the cathode side (Fig. 4c). During Li^+^ ion intercalation, anisotropic volume changes induce stress at grain boundaries, leading to crack initiation [44]. The AGG-dominated solvation structure in TFEE-LHCE exacerbates oxidative side reactions of the electrolyte on the particle surface, rendering intergranular crack formation more severe and irreversible. The CEI formed by TFEE-LHCE is approximately 8 nm thick, further confirming more intensive oxidative reactions at the cathode interface, resulting in a high-impedance CEI layer (Fig. 4f).

XPS analysis of the chemical composition of the cycled CEI mainly detects C–C/C–H (284.8 eV) and C–O (289.1 eV) peaks from organic decomposition products, as well as inorganic components such as LiF (684.5 eV) from the decomposition of FSI^−^ anions. Consistent with the SEI composition trend, the signal intensities of LiF and organic species in the TFEE-LHCE system exhibit a more pronounced vertical distribution with increasing sputtering time (Fig. 4g, h). This indicates that the electron-rich AGG structure in TFEE-LHCE undergoes significantly more severe oxidative decomposition compared to the CIP-dominated solvation structure in TFE-LHCE. Although LiF, as an electronic insulator, can provide protection to the cathode, its ionic conductivity is relatively low. The high-impedance CEI layer and the continuous decomposition of anion-enriched AGGs collectively contribute to capacity decay and reduced cycle life in high-voltage LMBs.

### Electrochemical Performance and Practical Pouch Cell Validation

**Fig. 5:**
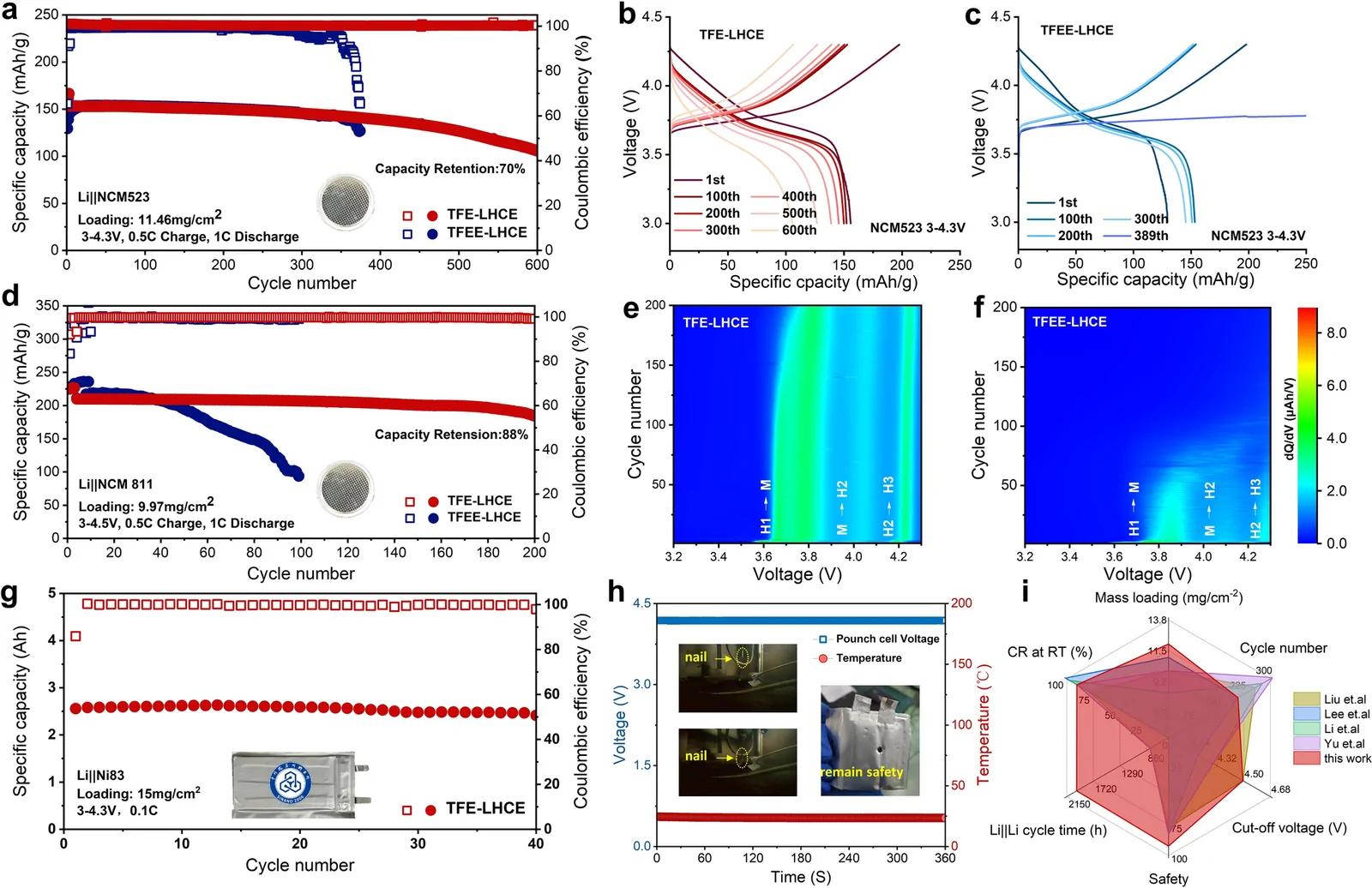
**a** Cycling performance and corresponding charge–discharge profiles of 4.3 V Li||NCM523 cells using **b** TFE-LHCE and **c** TFEE-LHCE electrolytes. **d** Cycling performance and corresponding dQ/dV profiles of Li||NCM811 cells using **e** TFE-LHCE and **f** TFEE-LHCE electrolytes. **g** Cycling performance of 2.6 Ah, 4.3 V Li||Ni83 pouch cells using TFE-LHCE electrolyte at 0.1 C. **h** Nail penetration tests of fully charged 2.6 Ah Li||NCM83 pouch cell with TFE-LHCE. The insets are the optical images of test progress. **i** Performance radar plot of TFE-LHCE versus recently reported ionic liquid-based electrolytes

We further validated the performance differences between the two electrolyte systems using coin cell and pouch cell, and assessed the application potential and safety of the TFE-LHCE electrolyte in Ah-level pouch cells. First, Li||NCM523 (LiNi_0.5_Mn_0.3_Co_0.2_O_2_) cells were assembled using a high-mass-loading cathode (11.46 mg cm^−2^). As shown in Fig. 5a-c, under a cutoff voltage of 4.3 V and charge/discharge rates of 0.5 C/1 C, the Li||TFE-LHCE||NCM523 cell exhibited stable cycling for 600 cycles with a capacity retention of 70%. In contrast, the Li||TFEE-LHCE||NCM523 cell showed rapid capacity and coulombic efficiency decay after approximately 380 cycles. When using a higher-nickel-content NCM811 cathode and increasing the cutoff voltage to 4.5 V, the Li||TFE-LHCE||NCM811 cell achieved stable cycling for 200 cycles under 0.5 C charge/discharge conditions with a capacity retention of 88%. By comparison, the Li||TFEE-LHCE||NCM811 cell exhibited rapid capacity degradation from the early cycling stages (Figs. 5d and S17). The differential capacity (dQ/dV) curves during the charging process of Li||NCM811 cells were analyzed (Fig. 5e, f). The three characteristic peaks located at approximately 3.7, 4.0, and 4.2 V correspond to the three phase transition stages of Li^+^ intercalation into the NCM811 cathode. The Li||TFE-LHCE||NCM811 cell maintained stable peak positions and intensities throughout cycling without significant shifts or attenuation, indicating highly reversible phase transitions. In contrast, the Li||TFEE-LHCE||NCM811 cell exhibited notable peak shifts and intensity reductions from the initial cycles, suggesting poor reversibility of Li^+^ intercalation reactions and severe structural degradation of the cathode material. The rate capability test for Li||NCM811 cells was also performed. As shown in Fig. S18, the cell with TFE-LHCE demonstrates superior capacity retention at high current densities (e.g., 1.3 C, 1.5 C) compared to TFEE-LHCE. This is a direct consequence of the faster Li⁺ transport kinetics and lower interfacial resistance enabled by the CIP-dominated solvation structure.

Furthermore, we assembled a 2.6 Ah Li||TFE-LHCE||LiNi_0.83_Mn_0.1_Co_0.07_O_2_ (Ni83) pouch cell. Under 0.1 C charge/discharge conditions, the cell demonstrated stable cycling for 40 cycles with a capacity retention of 95% (Figs. 5g and S19). Nail penetration tests showed that the Li||TFE-LHCE||Ni83 pouch cell did not ignite or explode during the entire process, demonstrating excellent safety performance (Fig. 5h). The radar chart in Fig. 5i further illustrates that the TFE-LHCE system outperforms several reported ionic liquid-based electrolyte systems in key performance metrics, including cycle life, voltage tolerance, LMAs compatibility, and safety [45–49].

## Conclusions

In summary, we propose a novel anion–diluent decoupled solvation structure, which minimizes the transfer of negative charge from FSI^−^ anions to diluent molecules, thereby promoting anion–diluent complex decoupling and inducing a CIP-dominated solvation. Through molecular dynamics simulations, Fourier transform spectroscopy, and Raman spectroscopy tests, we confirmed diluent which have weak ion–dipole and hydrogen-bonding interactions between the diluent and FSI^−^ could promote coordination between Li^+^ ions and FSI^−^ anions, leading to the formation of a CIP-dominated solvation structure. The electrolyte with this CIP-dominated solvation structure demonstrates superior Li^+^ ion transport kinetics and enhanced oxidation resistance. Based on this principle, we designed the TFE-LHCE electrolyte, TFE not only promotes the formation of dense Li metal morphology at the anode but also suppresses electrolyte oxidation at the cathode. Using this electrolyte, Li||NCM523 coin cells exhibited 70% capacity retention after 600 cycles at 4.3 V, while Li||NCM811 coin cells achieved 88% capacity retention after 200 cycles at 4.5 V. Furthermore, a 2.6 Ah Li||Ni83 pouch cell demonstrated stable cycling for over 40 cycles and passed rigorous nail penetration tests. This study elucidates the mechanism by which non-solvating inert diluents reconfigure the Li^+^ ion solvation structure, establishing a theoretical foundation for designing high-performance and high-safety electrolytes.

## Supplementary Information

Below is the link to the electronic supplementary material.

Supplementary file1 (DOC 5118 KB)
