# Supplementary material for: Anion–Diluent Decoupled Solvation Chemistry in Ionic Liquid-Based Localized High-Concentration Electrolytes Toward High-Voltage Lithium Metal Batteries
Source: Nanomicro Lett. 2026 Jun 1;18:394. doi: 10.1007/s40820-026-02242-4 (PMC13226750; doi:10.1007/s40820-026-02242-4)
Supplement: Supplementary file 1 — Supplementary file1 (DOC 5118 KB) [file 40820_2026_2242_MOESM1_ESM.docx]

Supporting Information for

**Anion-Diluent Decoupled Solvation Chemistry in Ionic Liquid-Based Localized High-Concentration Electrolytes Toward High-Voltage Lithium Metal Batteries**

Guangye Wu^1,2+^, Haifeng Tu^2,3+^, Zhicheng Wang^4,5^*, Yiwen Gao^1,2^, Peng Ding^2,3^, Yi Yang^6^, Lingwang Liu^2,3^, Suwan Lu^2,3^, Farwa Mushtaq^2,3^, Guochao Sun^2,3^, Hexiang Chen^2,3^, Haiyang Zhang^2,3^, Jiangyan Xue^2,3^, Jingjing Xu^6^, Hong Li^4,5^, and Xiaodong Wu^2,3,4^*

^1^School of Materials Science and Engineering, Shanghai University, Shanghai 200444, P. R. China

^2^*i*-lab, Suzhou Institute of Nano-Tech and Nano-Bionics, Chinese Academy of Sciences, Suzhou, Jiangsu 215123, P. R. China

^3^School of Nano-Tech and Nano-Bionics, University of Science and Technology of China, Hefei, Anhui 230026, P. R. China

^4^Tianmu Lake Institute of Advanced Energy Storage Technologies Co., Ltd., Liyang 213300, P. R. China

^5^Beijing Advanced Innovation Center for Materials Genome Engineering Key Laboratory for Renewable Energy, Beijing Key Laboratory for New Energy Materials and Devices, Institute of Physics, Chinese Academy of Sciences, Beijing 100190, P. R. China

^6^College of Material Science and Engineering, Hohai University, Changzhou, Jiangsu 213022, P. R. China

^+^Guangye Wu and Haifeng Tu contributed equally to this work.

*Corresponding authors. E-mail: [zc_wang2023@163.com](mailto:zc_wang2023@163.com) (Zhicheng Wang); [xdwu2011@sinano.ac.cn](mailto:xdwu2011@sinano.ac.cn) (Xiaodong Wu)


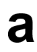
**Supplementary Figures and Tables**

**
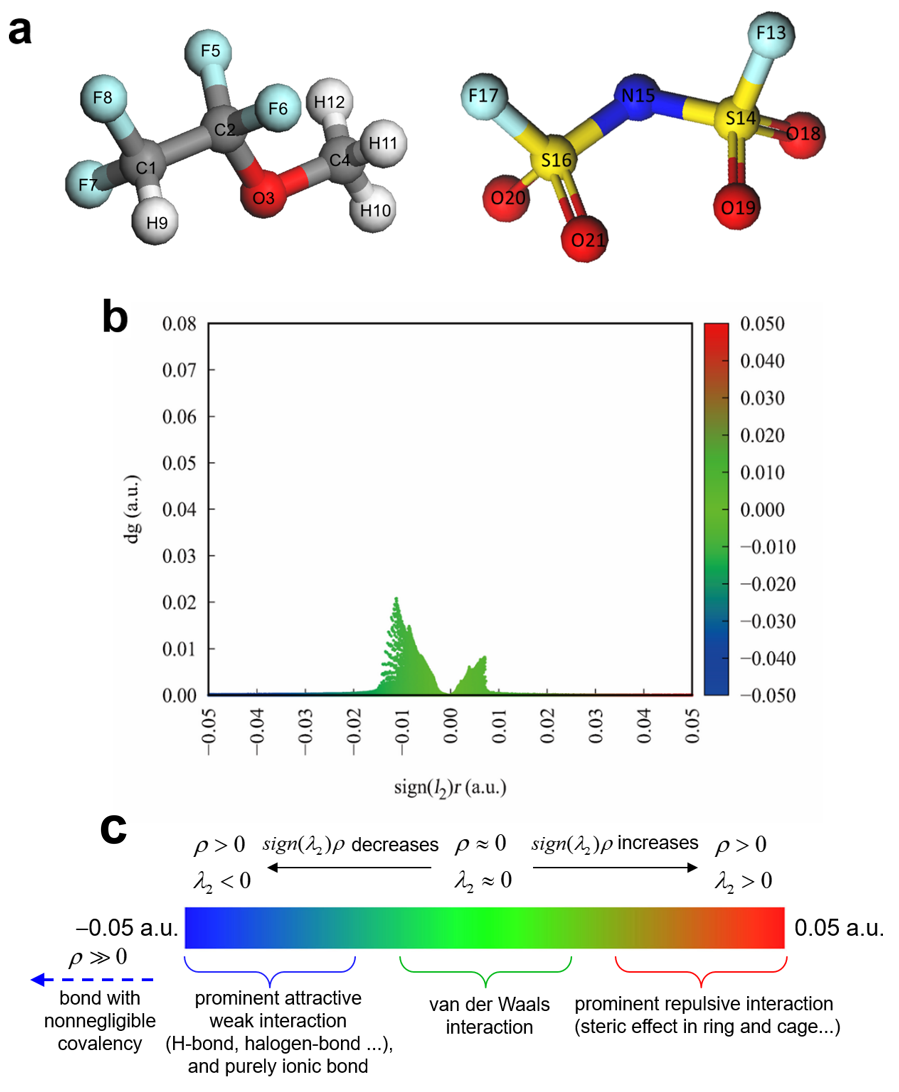
**

**Fig. S1** (a) Reference labels for atoms in the intermolecular contact matrix of TFE and FSI^-^ anion. (b) Scatter map between RDG and *sign(I_2_)r* of the grid points used to Fig. 1c. (c) Commonly used color scale in non-covalent interaction (NCI) map and common interpretation of various color ranges.

**
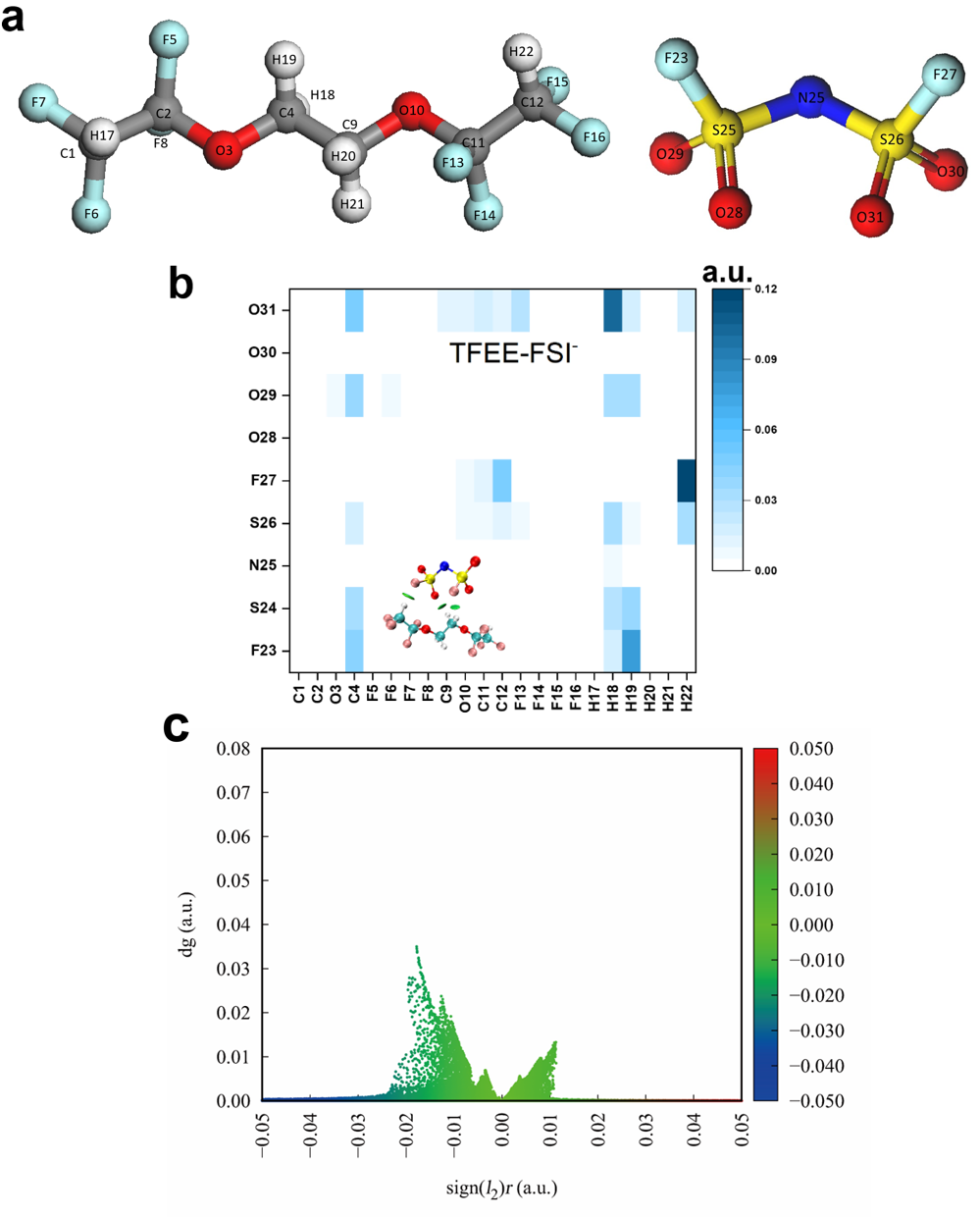
**

**Fig. S2** (a) Reference labels for atoms in the intermolecular contact matrix of TFEE and FSI^-^ anion. (b) Intermolecular contact matrix and interaction energy gradient isosurfaces of TFEE-FSI^-^ obtained by the RDG (reduced density gradient) method. (c) corresponding scatter plot between RDG and sign*(I_2_)r*.


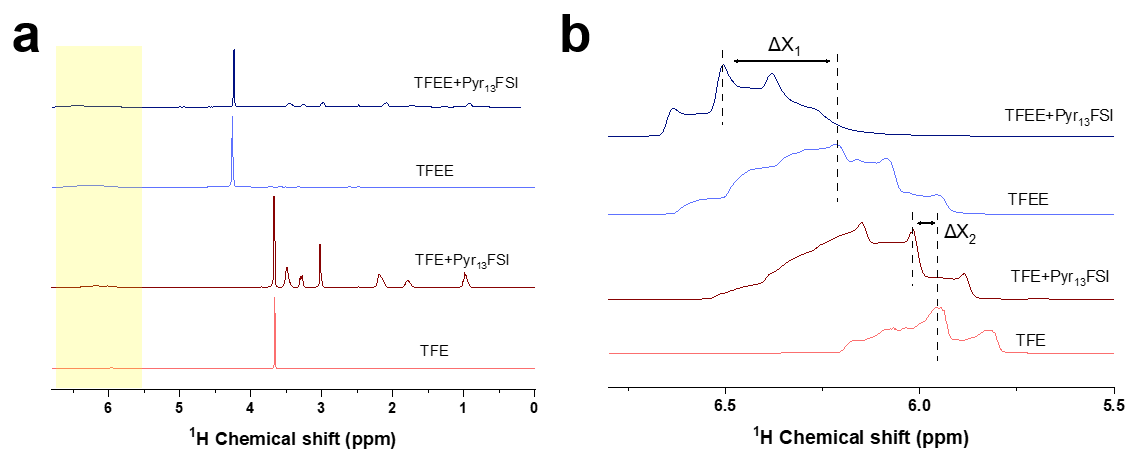


**Fig. S3** (a, b) The ¹H NMR spectra of the pure diluent and the diluent after being mixed with the ionic liquid.


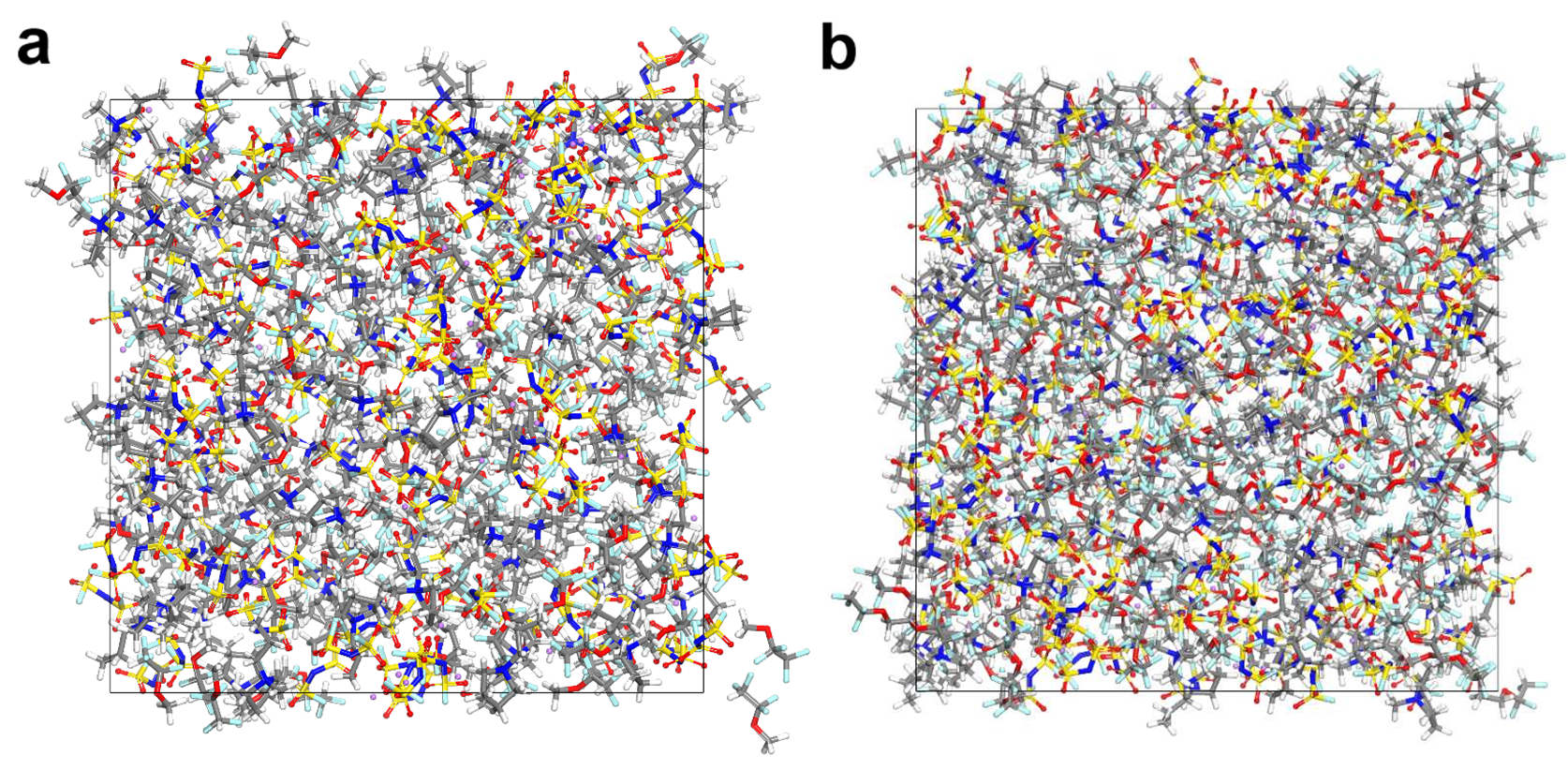


**Fig. S4** Molecular dynamics (MD) simulation snapshot of (a) TFE-LHCE and (b) TFEE-LHCE.


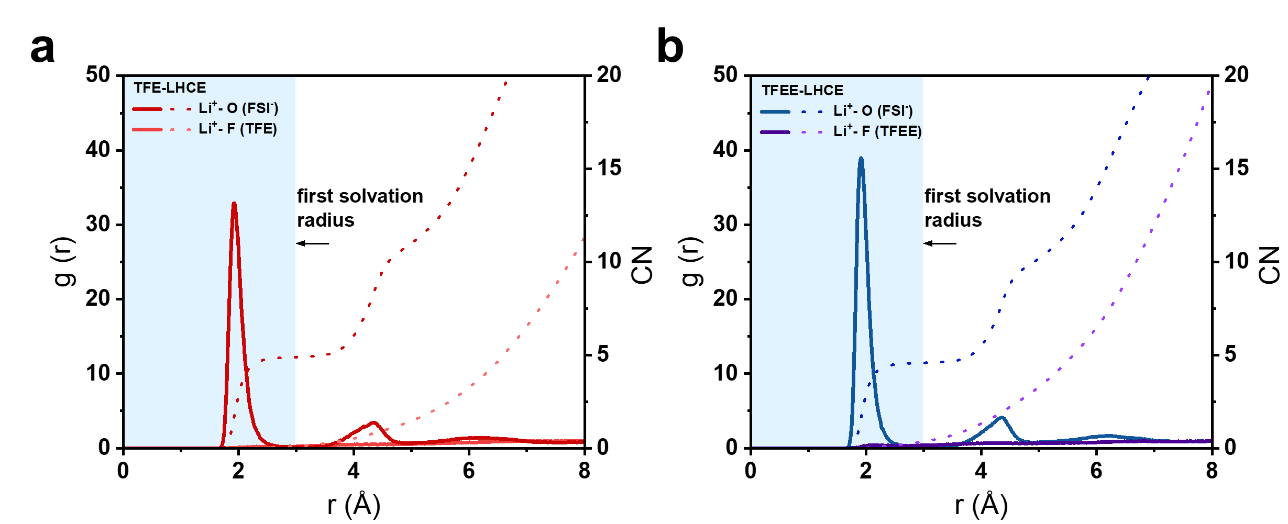


**Fig. S5** (a) RDF and CN of Li^+^-O (FSI^-^) and Li^+^-F (TFE) obtained from MD simulations in TFE-LHCE. (b) RDF and CN of Li^+^-O (FSI^-^) and Li^+^-F (TFEE) obtained from MD simulations in TFEE-LHCE.


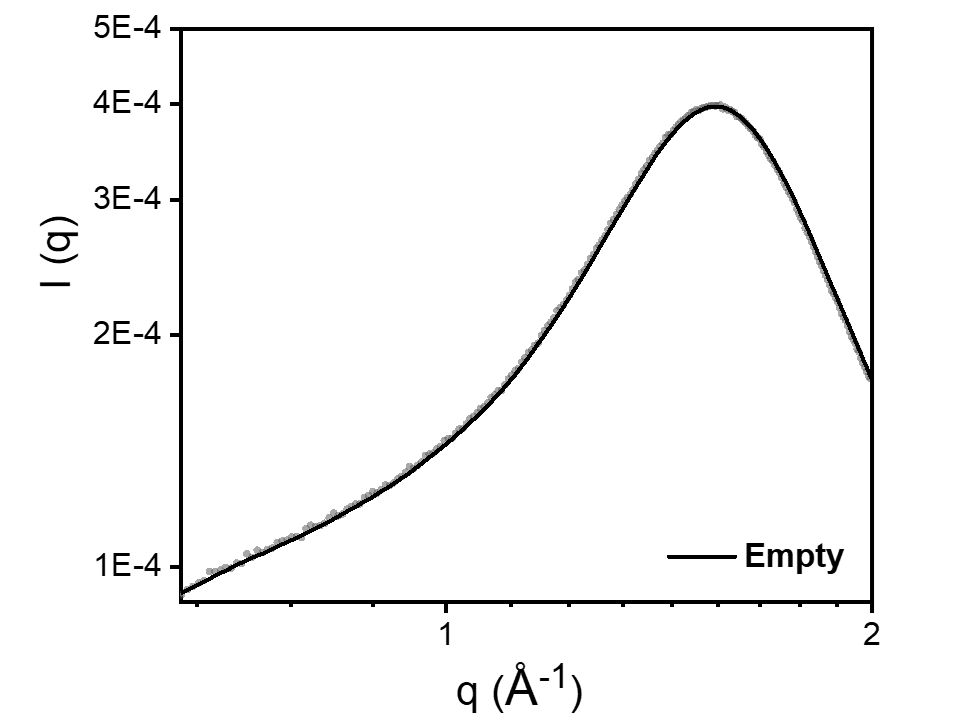


**Fig. S6** Wide-angle X-ray scattering (WAXS) test result of an empty glass capillary.


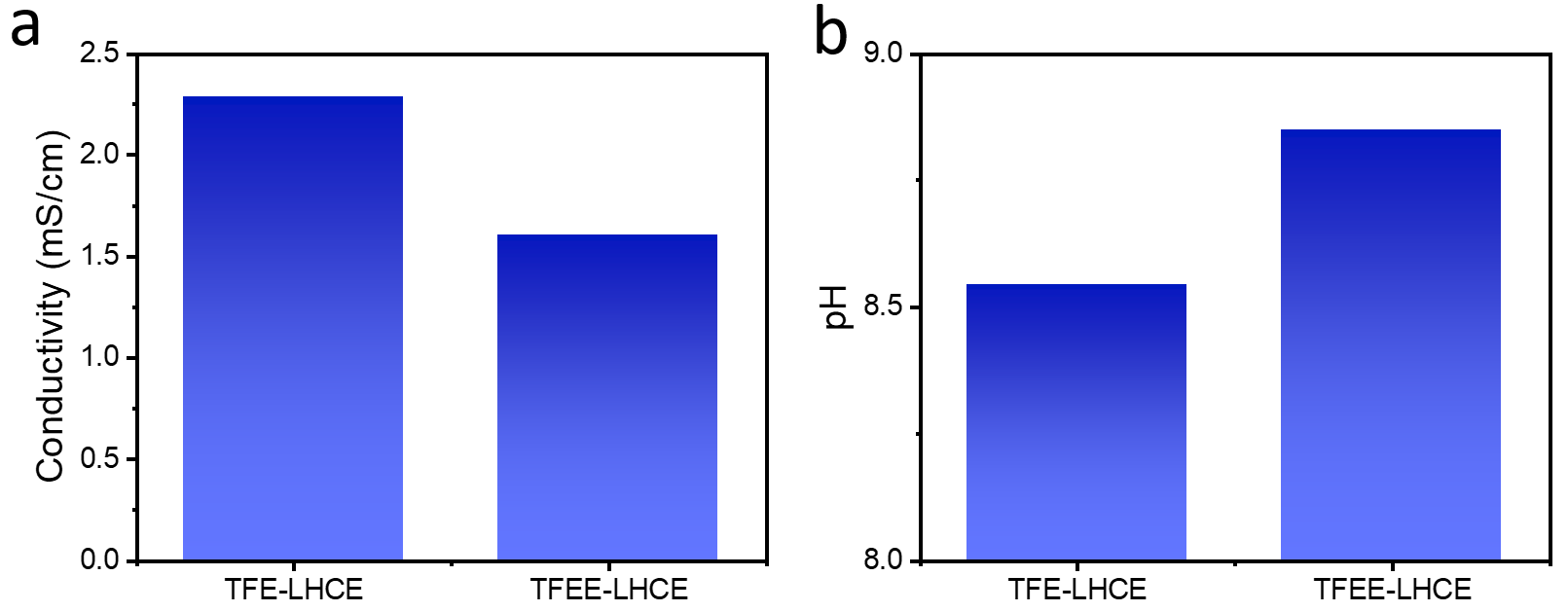


**Fig. S7** (a) Ionic conductivities and (b) pH value of TFE-LHCE and TFEE-LHCE were tested by using an Mettler Toledo S400.


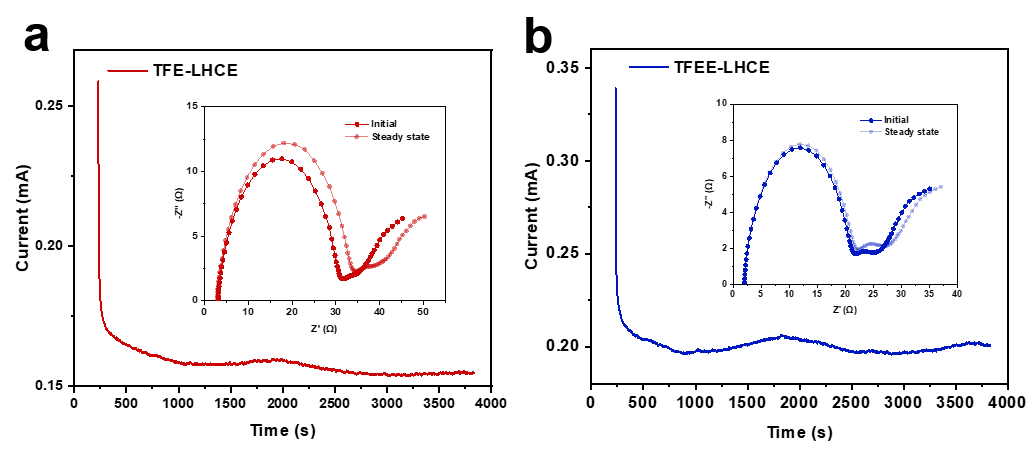


**Fig. S8** (a, b) The lithium-ion transference numbers of the two electrolytes were determined via direct current polarization combined with alternating current impedance using Li||Li symmetric cells.

**Fig. S9** The viscosities of the LiFSI-Pyr_13_FSI electrolyte, TFE-LHCE electrolyte, and TFEE-LHCE electrolyte.


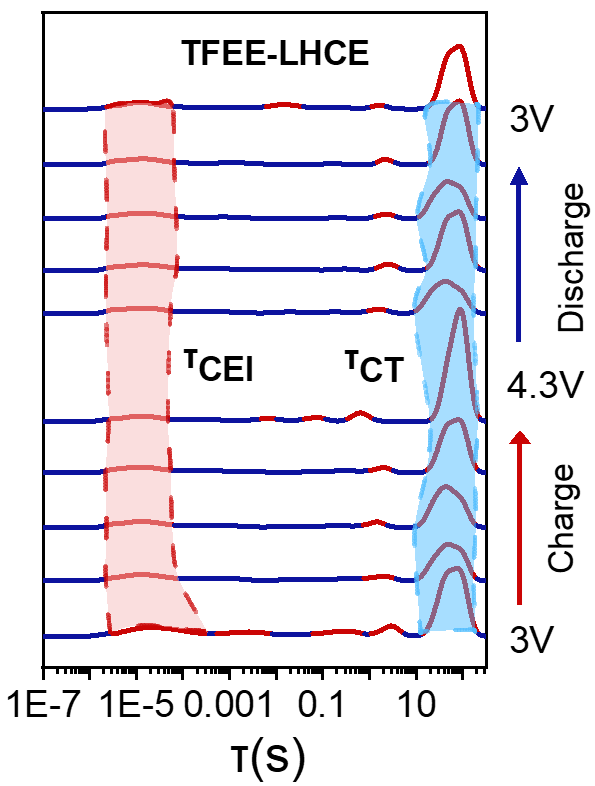


**Fig. S10** Relaxation Time Distribution (DRT) derived from in-situ electrochemical impedance spectroscopy (EIS) of Li||TFEE-LHCE||NCM811 cells after formation cycles at a 4.3 V cutoff voltage.

**Fig. S11** DRT analysis derived from in-situ EIS of (a) Li|TFE-LHCE|Li and (b) Li|TFEE-LHCE|Li cells after formation cycles.


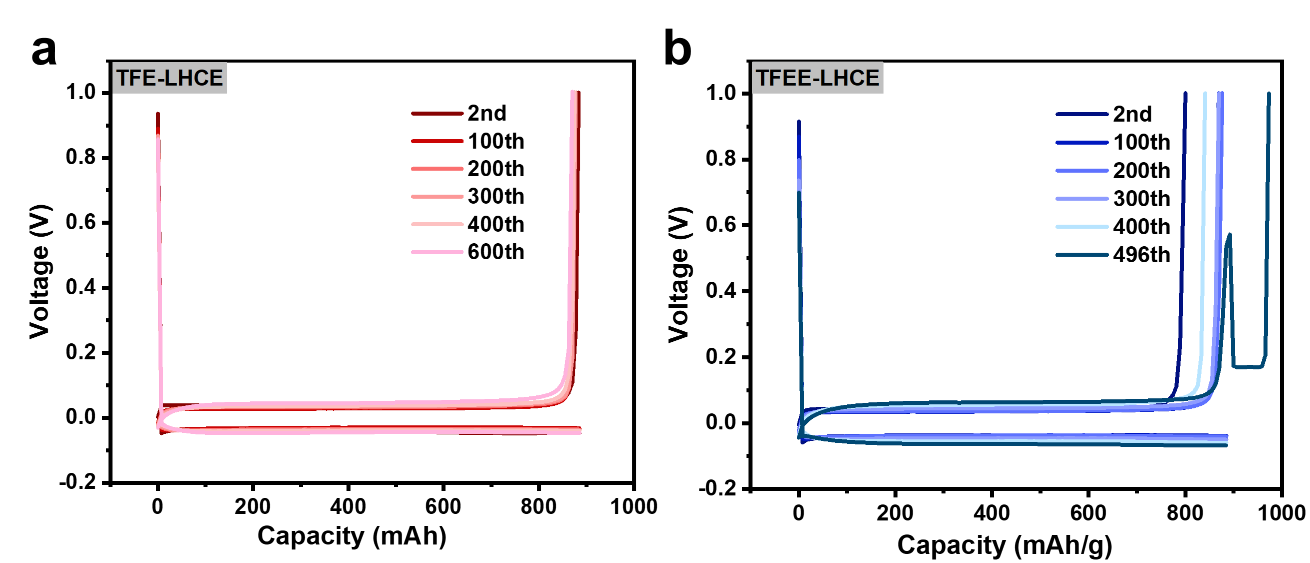


**Fig. S12** Li plating/stripping voltage profiles in Li||Cu cells with (a) TFE-LHCE and (b) TFEE-LHCE.


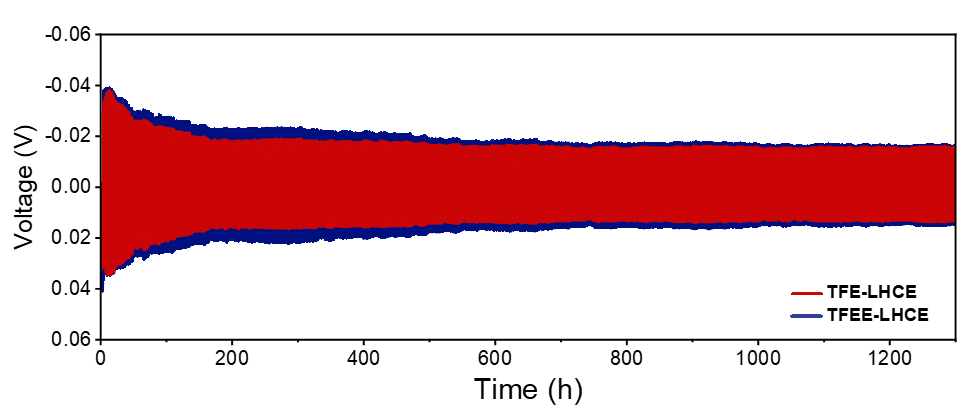


**Fig. S13** Cycling performance of the Li||Li cell at 0.1 mA cm^-2^ and 0.1 mAh cm^-2^.


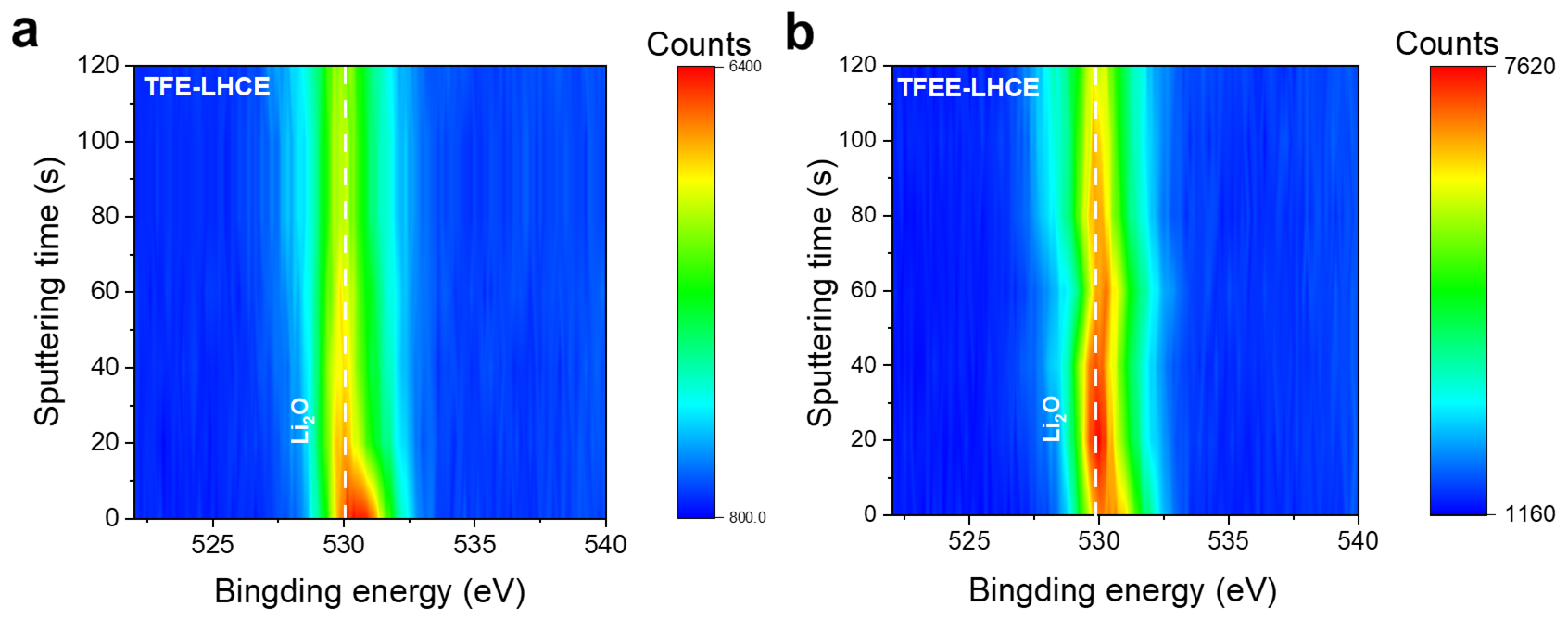


**Fig. S14** XPS O1s contour plots of lithium metal after 50 cycles with (a) TFE-LHCE and (b) TFEE-LHCE electrolytes.


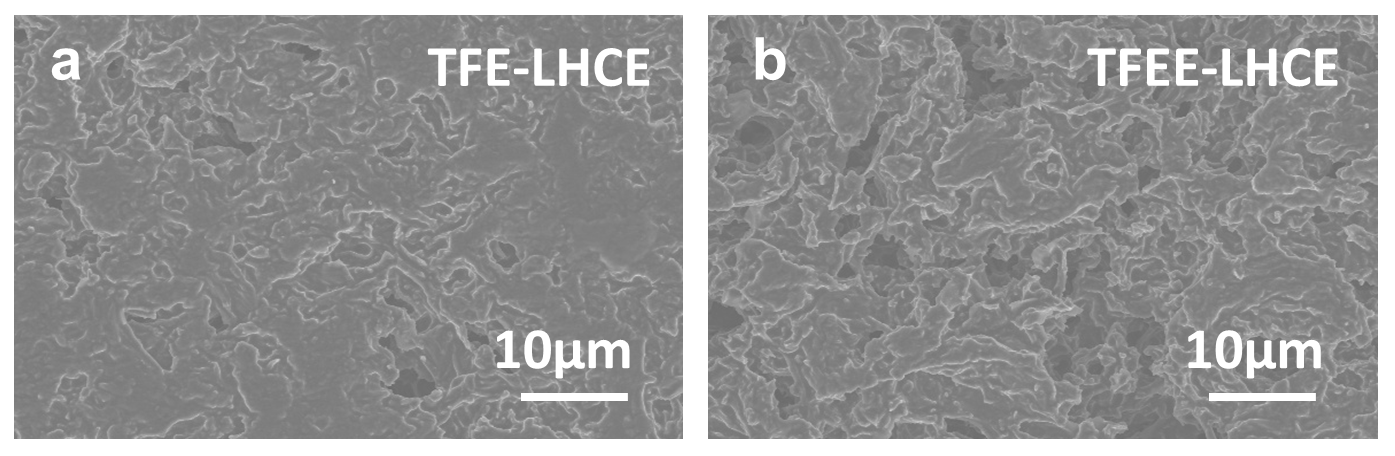


**Fig. S15** Top-view SEM morphologies of lithium metal after cycling in (a) Li||TFE-LHCE||Li and (b) Li||TFEE-LHCE||Li.


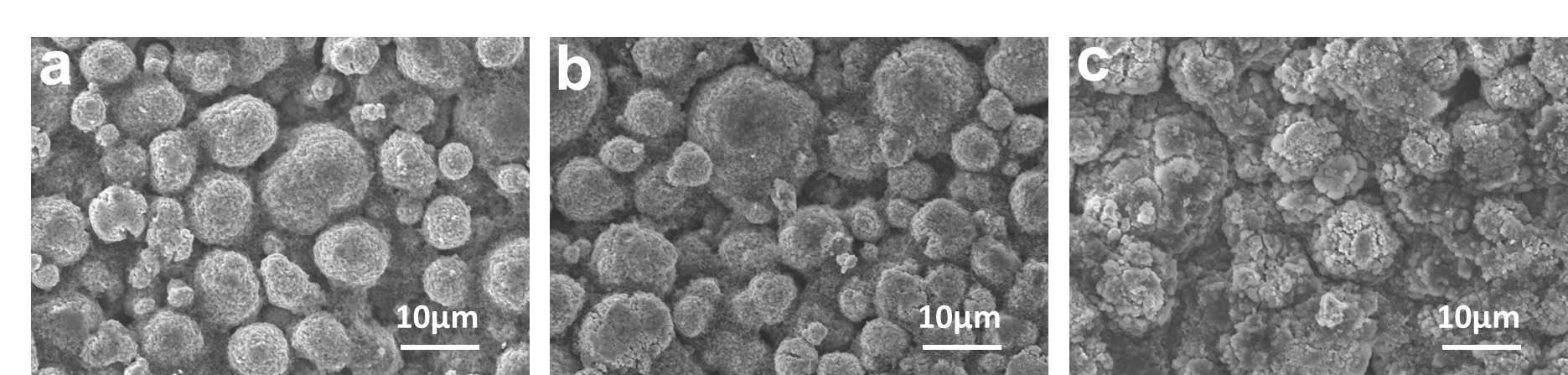


**Fig. S16** Top-view SEM morphologies of (a) pristine NCM811 cathode and after 200 cycles with (b) TFE-LHCE and (c) TFEE-LHCE.


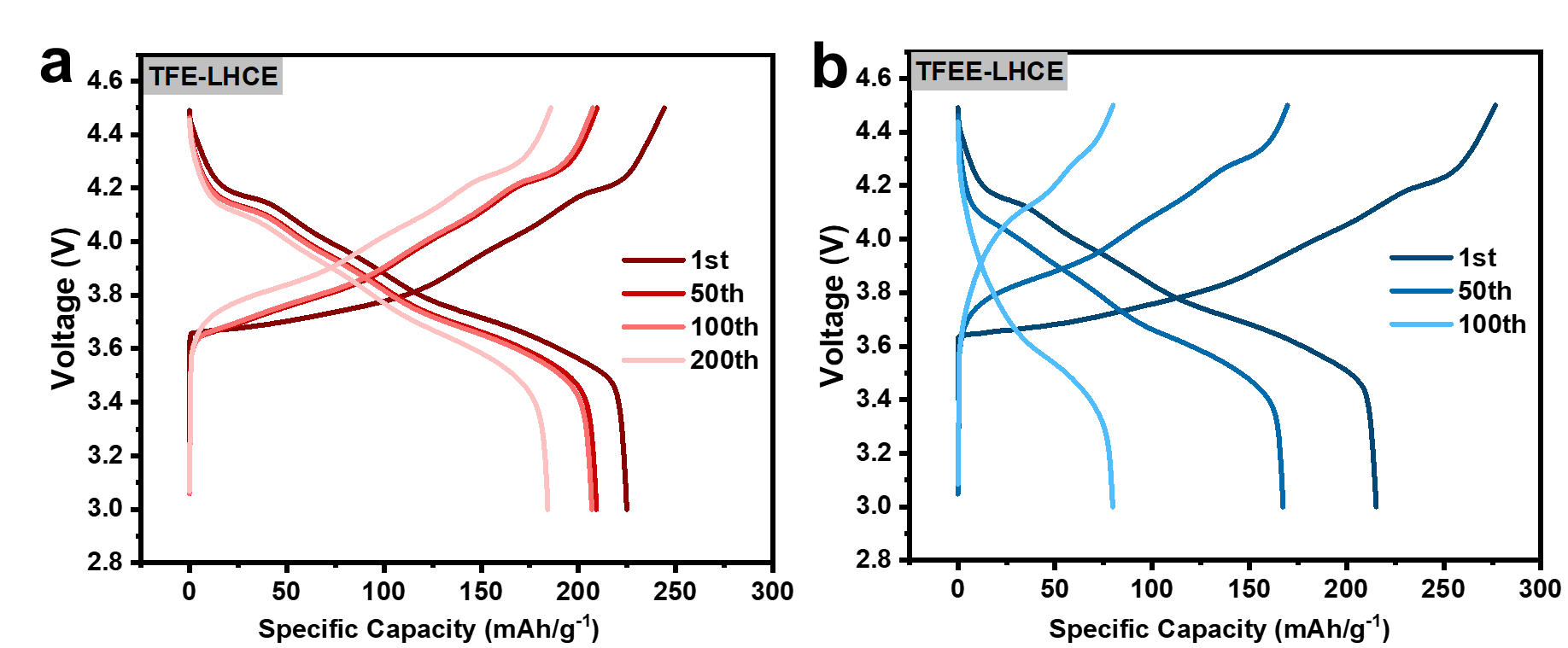


**Fig. S17** Charge/discharge voltage profiles of (a) Li||TFE-LHCE||NCM811 and (b) Li||TFEE-LHCE||NCM811 coin cell under 3.0-4.3 V at 0.5 C.

**Fig. S18** The rate capability of the Li||NCM811 batteries with different electrolytes.


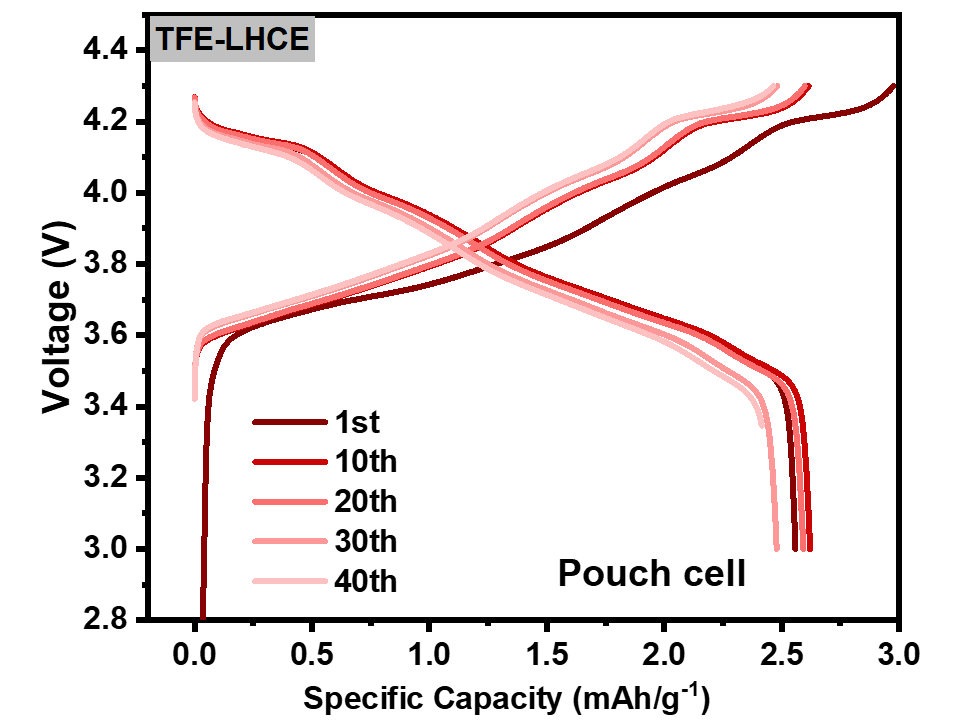


**Fig. S19** Charge/discharge voltage profiles of 2.6 Ah Li||TFE-LHCE||Ni83 pouch cell under 3.0-4.3 V at 0.1 C.
